# Targeting CXCR4/CXCL12 axis via [^177^Lu]Lu-DOTAGA.(SA.FAPi)_2_ with CXCR4 antagonist in triple-negative breast cancer

**DOI:** 10.1007/s00259-024-06704-y

**Published:** 2024-04-08

**Authors:** Guangfa Bao, Ziqiang Wang, Luoxia Liu, Buchuan Zhang, Shuang Song, Dongdong Wang, Siyuan Cheng, Eu-Song Moon, Frank Roesch, Jun Zhao, Bo Yu, Xiaohua Zhu

**Affiliations:** 1 Department of Nuclear Medicine, Tongji Hospital, Tongji Medical College, Huazhong University of Science and Technology, 1095 Jiefang Ave, Wuhan, 430030 China; 2 Department of Chemistry, Johannes Gutenberg University, 55131 Mainz, Germany; 3 Department of Anatomy, School of Basic Medicine, Huazhong University of Science and Technology, Wuhan, 430030 Hubei Province China; 4 Cell Architecture Research Center, Huazhong University of Science and Technology, Wuhan, Hubei Province 430030 China

**Keywords:** [^177^Lu]Lu-DOTAGA.(SA.FAPi)_2_, CXCR4 antagonist, CXCR4/CXCL12, Triple-negative breast cancer (TNBC)

## Abstract

**Purpose:**

Radiopharmaceutical therapies targeting fibroblast activation protein (FAP) have shown promising efficacy against many tumor types. But radiopharmaceuticals alone in most cases are insufficient to completely eradicate tumor cells, which can partially be attributed to the protective interplay between tumor cells and cancer-associated fibroblasts (CAFs). The C-X-C chemokine receptor type 4/C-X-C motif chemokine 12 (CXCR4/CXCL12) interaction plays an important role in orchestrating tumor cells and CAFs. We hereby investigated the feasibility and efficacy of [^177^Lu]Lu-DOTAGA.(SA.FAPi)_2_, a FAP-targeting radiopharmaceutical, in combination with AMD3100, a CXCR4 antagonist, in a preclinical murine model of triple-negative breast cancer (TNBC).

**Methods:**

Public database was first interrogated to reveal the correlation between CAFs’ scores and the prognosis of TNBC patients, as well as the expression levels of FAP and CXCR4 in normal tissues and tumors. In vitro therapeutic efficacy regarding cell proliferation, migration, and colony formation was assessed in BALB/3T3 fibroblasts and 4T1 murine breast cancer cells. In vivo therapeutic efficacy was longitudinally monitored using serial ^18^F-FDG, [^18^F]AlF-NOTA-FAPI-04, and [^68^Ga]Ga-DOTA-Pentixafor PET/CT scans and validated using tumor sections through immunohistochemical staining of Ki-67, α-SMA, CXCR4, and CXCL12. Intratumoral abundance of myeloid-derived suppressive cells (MDSCs) was analyzed using flow cytometry in accordance with the PET/CT schedules. Treatment toxicity was evaluated by examining major organs including heart, lung, liver, kidney, and spleen.

**Results:**

CAFs’ scores negatively correlated with the survival of TNBC patients (*p* < 0.05). The expression of CXCR4 and FAP was both significantly higher in tumors than in normal tissues. The combination of [^177^Lu]Lu-DOTAGA.(SA.FAPi)_2_ and AMD3100 significantly suppressed cell proliferation, migration, and colony formation in cell culture, and exhibited synergistic effects in 4T1 tumor models along with a decreased number of MDSCs. PET/CT imaging revealed lowest tumor accumulation of ^18^F-FDG and [^18^F]AlF-NOTA-FAPI-04 on day 13 and day 14 after treatment started, both of which gradually increased at later time points. A similar trend was observed in the IHC staining of Ki-67, α-SMA, and CXCL12.

**Conclusion:**

The combination of [^177^Lu]Lu-DOTAGA.(SA.FAPi)_2_ and AMD3100 is a feasible treatment against TNBC with minimal toxicity in main organs.

**Supplementary Information:**

The online version contains supplementary material available at 10.1007/s00259-024-06704-y.

## Introduction

Patients with triple-negative breast cancer (TNBC) suffer from poor prognosis due to limited therapeutic options, low response rate, and high relapse rate [1]. One possible mechanism for this refractory disease lies in the coordinated interplay between tumor cells and cancer-associated fibroblasts (CAFs), including that through the C-X-C chemokine receptor type 4/C-X-C motif chemokine 12 (CXCR4/CXCL12) signaling [2, 3]. Indeed, over 75% of TNBC tumors highly express CXCR4, while fibroblast activation protein (FAP)-positive CAFs are found to be the only stromal source of CXCL12 [4–6]. The activation of CXCR4/CXCL12 signaling can further stimulate its downstream phosphatidylinositol-4,5‑bisphosphate 3‑kinase (PI3K)/ protein kinase B (PKB/AKT) and mitogen-activated protein kinases (MAPK) signaling pathways, leading to protumoral changes such as angiogenesis, extracellular matrix remodeling, establishment of pre-metastatic niche, recruitment of CXCR4^+^ tumor cells, and polarization of the immune microenvironment toward an immunosuppressive one [7, 8]. Therefore, the CXCR4/CXCL12 axis is a potential target for TNBC therapy.

The disruption of CXCR4/CXCL12 signaling can enhance the efficacy of chemotherapy and immunotherapy by blunting the migration and metastasis of tumor cells and remodeling of the immunosuppressive tumor microenvironment (TME) [9]. Several CXCR4 antagonists have been approved by the Food and Drug Administration (FDA) of the USA, such as AMD3100 (commercially branded as plerixafor) [10], LY2510924 [11], balixafortide [9], and BL-8040 [12]. The combination of balixafortide and eribulin elevated the 1-year survival rate of patients with HER2-negative breast cancer by 1.5-folds compared to that of eribulin monotherapy group [9]. BL-8040 treatment, in conjunction with pembrolizumab and chemotherapy, increased the tumor-infiltration of CD8^+^ effector T cells while decreasing the frequency of myeloid-derived suppressor cells (MDSCs) and circulating regulatory T cells in patients with metastatic pancreatic ductal adenocarcinoma [12]. AMD3100 was also evaluated along with radiotherapy against high-grade glioma in a clinical trial [13]. However, there has been no reports on the combination of radiopharmaceutical therapy (RPT) and CXCR4 antagonists in cancer therapy.

Fibroblast activation protein (FAP) targeted RPT could deliver radiation dose to CAFs, not only killing CAFs and adjacent TNBC tumor cells, but also reducing the secretion of CXCL12 by CAFs. Addition of CXCR4 antagonist would further disrupt the CXCR4/CXCL12 signaling within the TME and thereby alleviate immunosuppression. Therefore, we hypothesized that the combination of FAP-targeted RPT and CXCR4 antagonist would effectively treat TNBC by direct tumor killing and TME reprogramming. To test this hypothesis, we prepared DOTAGA.(SA.FAPi)_2_ for the delivery of therapeutic radionuclide lutetium-177 (^177^Lu) and firstly evaluated the resultant [^177^Lu]Lu-DOTAGA.(SA.FAPi)_2_ in conjunction with AMD3100 in TNBC mouse models. DOTAGA.(SA.FAPi)_2_ is derived from a bidentate FAP inhibitor (FAPI) with a remarkably long/adequate median whole body effective time compared to monomeric FAPI-04 (86.6 h vs. 14 h) and therefore is more suitable to deliver therapeutic radionuclides [14–16]. Tumor response was monitored via positron emission tomography/computed tomography (PET/CT) imaging. The dynamic changes in the MDSCs were analyzed using flowcytometry.

## Methods

### Bioinformatic analysis

Transcriptome data and clinical data of TNBC patients were obtained from The Cancer Genome Atlas (TCGA) database and Gene Expression Omnibus (GEO) database (Accession number = GSE58812). The Estimate the Proportion of Immune and Cancer cells (EPIC), Microenvironment Cell Populations-counter (MCP-counter) algorithms, and Tumor Immune Dysfunction and Exclusion (TIDE) website (http://tide.dfci.harvard.edu) were applied to calculate the cancer-associated fibroblast scores in dataset. Then, the relationship of CAFs’ scores with TNBC patients’ survival was evaluated using Cox regression model. The expression of CXCR4 and FAP in normal tissues and tumor tissues was appraised using R software (R version 4.2.3).

### Cell line

4T1 mouse breast cancer cells (a kind gift from Dr. Xiang Ma, College of Pharmacy, Tongji Medical College, Huazhong University of Science and Technology) were cultured in complete RPMI 1640 medium supplemented with 10% fetal bovine serum and 100 U/ml penicillin–streptomycin at 37 °C in a humidified atmosphere with 5% CO_2_. BALB/3T3 clone A31 fibroblasts were purchased from Procell Life Science&Technology Co. Ltd, China, and cultured in Dulbecco’s modified eagle medium (DMEM).

### Radiolabeling

The radiosynthesis of [^18^F]AlF-NOTA-FAPI-04 was performed following a previous report [17]. Briefly, [^18^F]fluoride was trapped by a Sep-Pak light QMA cartridge and eluted by 0.2 mL saline. Then, 50 nmol NOTA-FAPI-04 (Nanchang Tanzhen Bio Co., Ltd) and 40 nmol AlCl_3_ were added into the vial; the reaction pH was adjusted to 4 using acetate buffer. After reaction at 110 °C for 15 min, the product was purified by passing through an activated C18 Sep-Pak Light solid-phase extraction cartridge (WAT023501, Waters, the USA). The purified product was characterized using high-performance liquid chromatography equipped with a radio-detector (radio-HPLC). The product was eluted using a gradient mobile phase from 8% solvent B at 0 min to 28% solvent B at 20 min: solvent A = 0.1% trifluoroacetic acid (TFA) in ultrapure H_2_O; solvent B = acetonitrile.

[^68^Ga]Ga-DOTA-Pentixafor radiolabeling was performed according to previous protocols [18]. Briefly, 2 ml of 0.05 M aqueous HCl containing ^68^Ga was transferred into a reactor vial containing 20 nmol of DOTA-Pentixafor (Nanchang Tanzhen Bio Co., Ltd) and 500 µL 0.25 M sodium acetate. The mixture was heated at 95 °C for 15 min with nitrogen slowly bubbled through the solution. The crude product was purified by passing through a C18 Sep-Pak cartridge. The radiolabeling efficiency was examined using radio-thin-layer chromatography using citrate solvent (0.1 M, pH 4.0) or 1 M ammonium acetate/methanol (50/50, v/v) as the mobile phase.

The process of ^177^Lu-labeling of DOTAGA.(SA.FAPi)_2_ (University Mainz, Germany) was the same as described in our earlier research [19]. After adding 150 MBq [^177^Lu]LuCl_3_ (Chengdu Xinke Pharmaceutical Co. Ltd, China) and 10 nmol DOTAGA.(SA.FAPi)_2_ to the vial, the reaction pH was adjusted to 5 and temperature increased to 95 °C and kept for 15 min. The crude product was purified by C18 Sep-Pak cartridge and sterilized for subsequent application. [^68^Ga]Ga-DOTAGA.(SA.FAPi)_2_ was prepared similarly to that of [^177^Lu]Lu-DOTAGA.(SA.FAPi)_2_.

### Cell proliferation and migration detection

Cell proliferation was measured with 5-ethynyl-2′-deoxyuridine (EdU) staining. Briefly, 4T1 cells or co-culture preconditioned BALB/3T3 fibroblasts were seeded in 6-well plates overnight and allocated into 4 groups: vehicle, AMD3100 (10 µg/ml), [^177^Lu]Lu-DOTAGA.(SA.FAPi)_2_ (5 MBq), and the combination. After adding therapeutic reagent, 10 µM EdU was added, and immunofluorescent EdU (red) and Hoechst (blue) staining was performed on days 1, 3, and 5 after treatment.

Cell migration was measured using transwell chambers. Briefly, the BALB/3T3 fibroblasts (5 × 10^5^) were seeded in 24-well plates, and 4T1 cells (3 × 10^4^) were added into the apical chamber, and 1 ml of DMEM culture medium containing 10% FBS was added into the basolateral chamber of a 24‐well plate. Cells were treated as previously described, cultured for 16 h, fixed with methanol, and stained with crystal violet ammonium oxalate solution (G1062, Solarbio, China). Unmigrated 4T1 cells were wiped away using swab, and the numbers of cells passing through the transwell were counted and used as an index to evaluate the migrant ability of 4T1 cells.

### Colony formation assay

For colony formation assays, co-culture preconditioned BALB/3T3 fibroblasts subjected to different treatment were plated into the 6-well plates at a density of 300 cells/well and then cultured in complete culture medium at 37 °C for 14 days. After gently washed in PBS twice, cells were fixed with 2% paraformaldehyde and stained with 0.1% (w/v) crystal violet. The numbers of colonies were counted and analyzed.

### Animal models and treatment

All animal studies were conducted in accordance with the Guide for the Care and Use of Medical Laboratory Animals (Ministry of Health, China). 4T1 cells (1 × 10^5^) were subcutaneously inoculated at the right shoulder of 6-week-old BALB/c mice (SPF Biotechnology Co. Ltd, China). When tumors reached a volume of 50 mm^3^, all mice were randomized into the following: vehicle, AMD3100, [^177^Lu]Lu-DOTAGA.(SA.FAPi)_2_, and the combination therapy. AMD3100 was injected intraperitoneally at 5 mg/kg per injection, once per day for 2 weeks. [^177^Lu]Lu-DOTAGA.(SA.FAPi)_2_ was intravenously administered at 18.5 MBq on days 3 and 8. The tumor growth was monitored by vernier caliper and calculated as volume = (length × width^2^)/2.

### Imaging

[^68^Ga]Ga-DOTAGA.(SA.FAPi)_2_ PET/CT imaging on a PET/CT scanner (uBio-EXPLORER, United Imaging, China) and [^177^Lu]Lu-DOTAGA.(SA.FAPi)_2_ single photon emission computed tomography (SPECT) imaging on a clinical SPECT/CT scanner (Discovery 670Pro, GE Healthcare, the USA) with intermediate energy collimator were performed to evaluate the biodistribution of [^177^Lu]Lu-DOTAGA.(SA.FAPi)_2_. ^18^F-FDG, [^18^F]AlF-NOTA-FAPI-04, and [^68^Ga]Ga-DOTA-Pentixafor PET/CT imaging were also performed on 6 tumor-bearing mice before treatment to evaluate their capacity to image 4T1 breast cancer models. Then, 4 mice in each group were randomly selected and received serial ^18^F-FDG, [^18^F]AlF-NOTA-FAPI-04, and [^68^Ga]Ga-DOTA-Pentixafor PET/CT scans on days 13-15, 18-20, 23-25, 28-30, and 33-35. All mice are treated as predetermined treatment plan. Given the large tumoral volumes of vehicle- and AMD3100-treated mice, the PET/CT scans for these two groups on days 28-30 and 33-35 were not performed. Owing to the death during imaging, there were only 3 mice in both groups left for PET/CT imaging on days 28-30 and 33-35. Mice were first intravenously injected with 1.85 MBq ^18^F-FDG, [^18^F]AlF-NOTA-FAPI-04, or 3.7 MBq [^68^Ga]Ga-DOTA-Pentixafor, respectively. If required (^18^F-FDG imaging), fast the mice for at least 6-8 h. One hour after tracer injection, the mice were anesthetized with 5% isoflurane and fixed at prone position. The anesthesia was maintained with 1% isoflurane during which a 10-min whole-body static PET/CT scan was performed. Data were reconstructed via using an ordered subsets expectation maximization (OSEM) algorithm (4 OSEM iterations, resolution: 1.4 mmHD) with scatter, attenuation, and decay corrections. The maximum standardized uptake value (SUVmax) was quantified and assessed among all groups.

### Immunohistochemistry

Harvested tumors were fixed, embedded in paraffin, and cut into a 4-µm thick sections. The sections were then re-hydrated, subjected to antigen retrieval, blocked with 5% bovine serum albumin (BSA) in Tris-buffered saline with Tween-20 (TBST), and incubated with antibodies against α-SMA (1/200, bs-10196R, Bioss, China), Ki-67 (1/200, 12202, Cell Signaling Technology, the USA), CXCL12 (ER1902-35, Huabio, China), and anti-rabbit IgG horseradish peroxide. Positive staining was detected via a 3,3′-diaminobenzidine (DAB) reaction. The sections were then counterstained with hematoxylin and imaged on a bright-field microscope.

### Flow cytometry

Tumors were cut into pieces and digested in a mixture comprising collagenase IV, hyaluronidase, and DNase I in DMEM medium at 37 °C under constant shaking for 30 min. After lysing red blood cells and removing debris, the cell mixture was washed and resuspended in PBS supplemented with 2% FBS for further analyses. Cells were counted and blocked with anti-mouse CD16/32, and then stained with the antibodies (anti-CD11b, anti-Gr-1). Samples were fixed and analyzed on a CytoFLEX-3 cytometer.

### Toxicity analyses

Paraformaldehyde-fixed paraffin-embedded mouse organs on days 19 including heart, lung, liver, kidney, and spleen were stained with hematoxylin-eosin for evaluating toxicity.

### Statistical analysis

Results were presented as mean ± standard error (SD). Significant difference among multiple data sets was determined using one-way analysis of variance (ANOVA). Log‐rank test was used for Kaplan‐Meier survival analysis. A *p*‐value less than 0.05 was considered statistically significant. Data analysis was conducted using Prism GraphPad 9 (GraphPad Software Inc., San Diego, CA, USA).

## Results

### Bioinformatic analysis

**Fig. 1:**
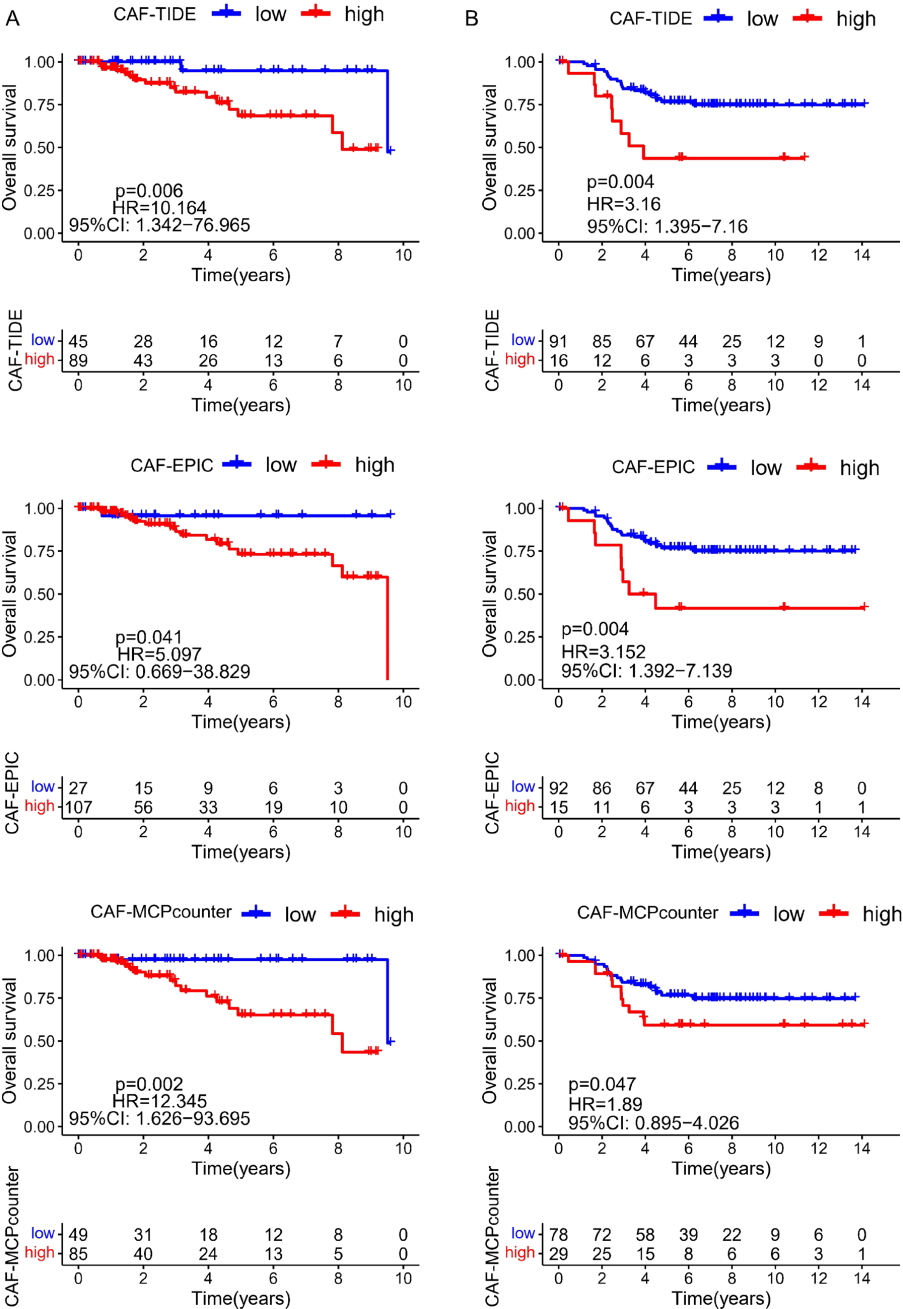
Correlation between the CAFs’ scores and the survival of TNBC patients. Data were generated from the TCGA (**A**, *n* = 134) and a GEO dataset (GSE58812-series) (**B**, *n* = 107). The CAFs’ scores were calculated using two R packages (CAF-EPIC, CAF-MCPcounter) and via a website (TIDE, http://tide.dfci.harvard.edu). Patients were then stratified into CAF high (red) or low (blue) groups using Cox regression model. Statistical parameters including *p* value, hazard rate (HR), and confidence interval (CI) are included in the figure

**Fig. 2:**
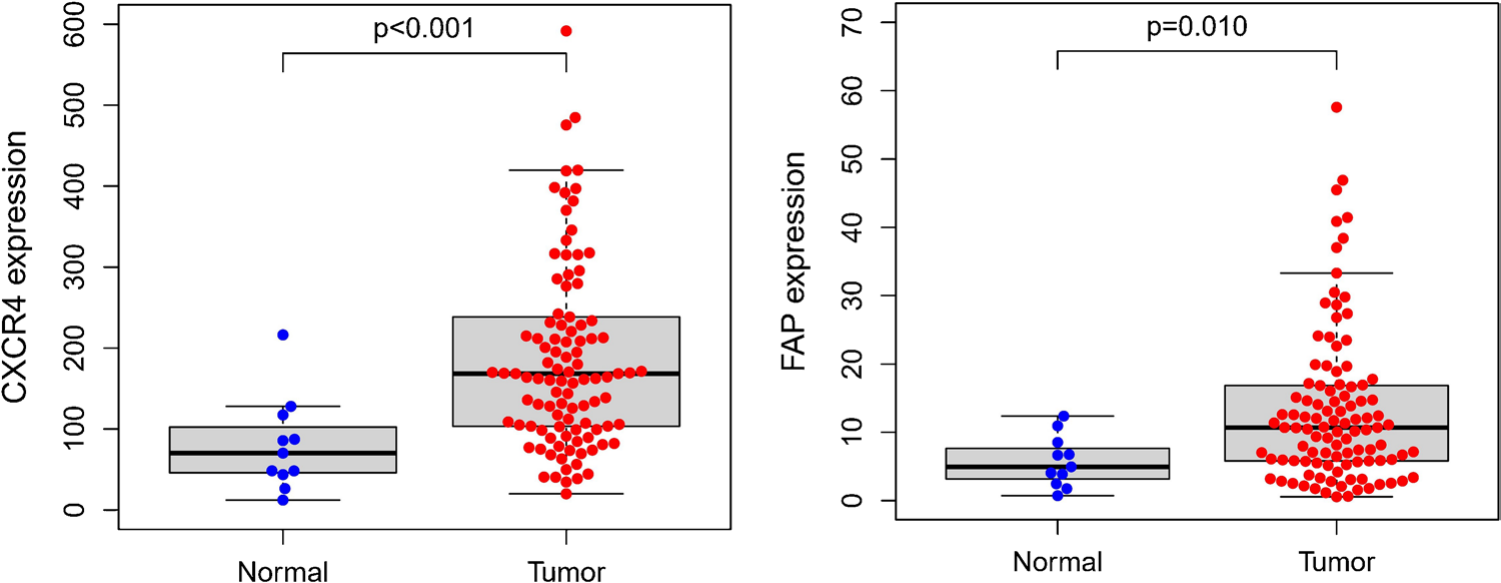
The gene expression of CXCR4 and FAP in normal tissues (*n* = 11) and TNBC tumors (*n* = 101). Data were obtained from the TCGA database and evaluated by R software; significance was determined using Student *t* test

TNBC patients were categorized into two groups according to the CAFs’ scores evaluated by different algorithms. Cox regression evaluation exhibited that the CAFs’ scores were negatively related to the TNBC patients’ survival (*p* < 0.05) (Fig. 1). Additionally, the expression of CXCR4 and FAP in tumors was significantly higher than that in normal tissues (CXCR4, *p* < 0.001; FAP, *p* = 0.010) (Fig. 2).

### Radiochemistry

The radiolabeling yield of [^177^Lu]Lu-DOTAGA.(SA.FAPi)_2_, [^68^Ga]Ga-DOTAGA.(SA.FAPi)_2_, and [^68^Ga]Ga-DOTA-Pentixafor were over 99%, and the radiochemical purity was over 95% after purification. In the solution of 1 M ammonium acetate methanol (50/50, v/v), both radiopharmaceuticals ([^177^Lu]Lu-DOTAGA.(SA.FAPi)_2_ and [^68^Ga]Ga-DOTA-Pentixafor) migrated with the solvent front, while free ^177^Lu^3+^ or ^68^Ga^3+^ remained at the origin. In the citrate solvent system, the radiolabeled pharmaceuticals remained at the origin, while the free ^177^Lu^3+^ or ^68^Ga^3+^ migrated in front of solvent (Supplemental Fig. 1A).

The retention time of [^18^F]AlF-NOTA-FAPI-04 was about 9 min in the HPLC chromatogram. There was no additional radioactive signal found in the HPLC analysis (Supplemental Fig. 1B).

### Cell proliferation, migration, and colony formation assay

**Fig. 3:**
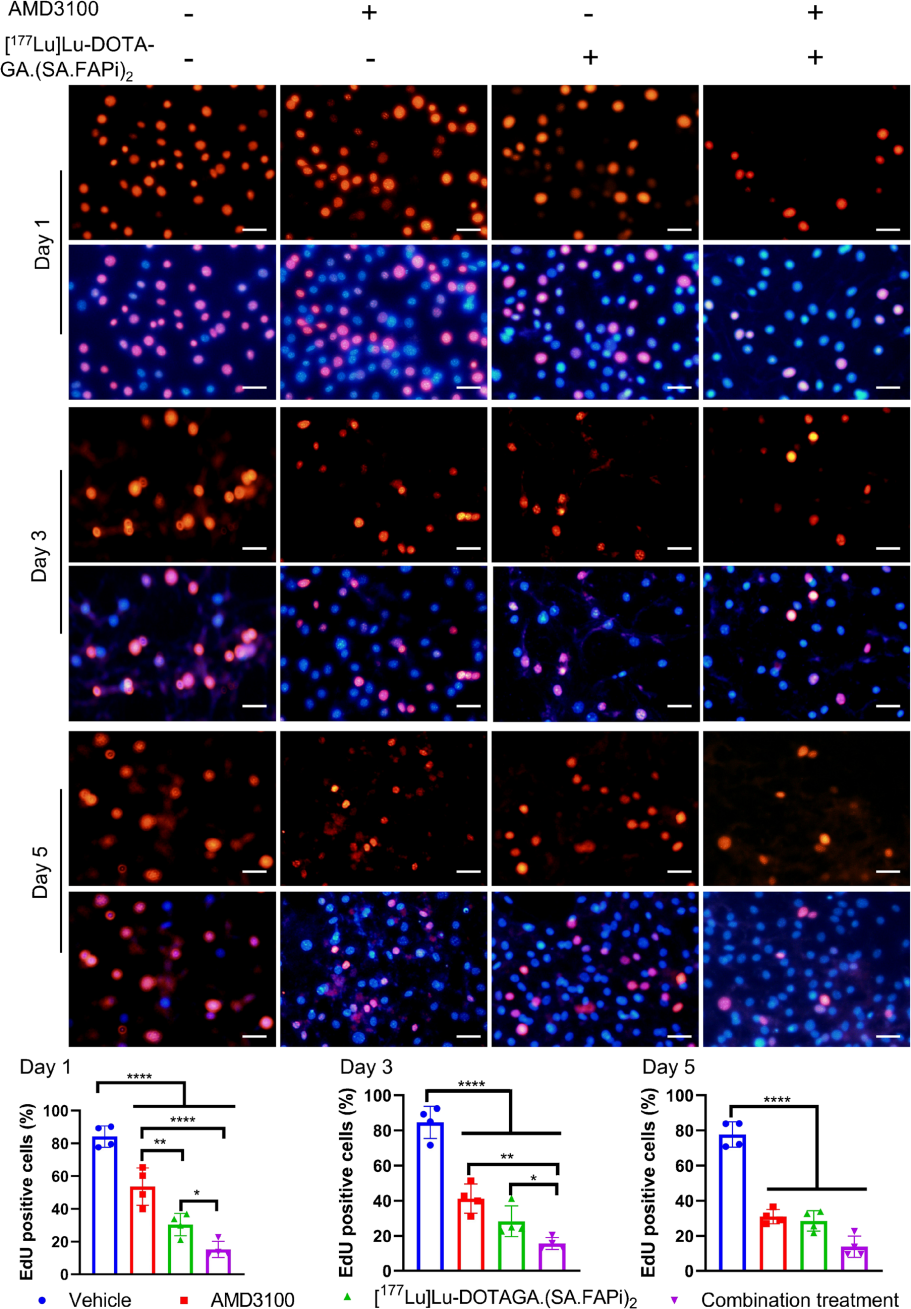
Effect of AMD3100 on BALB/3T3 fibroblast proliferation after [^177^Lu]Lu-DOTAGA.(SA.FAPi)_2_ treatment. Immunofluorescent EdU (red) and Hoechst (blue) staining of BALB/3T3 fibroblasts on days 1, 3, and 5 after treatment. Cells were non-treated, treated with [^177^Lu]Lu-DOTAGA.(SA.FAPi)_2_, AMD3100, or combination of both. Scale bar = 50 µm. The percentage of EdU positive cells were quantified and analyzed using one‐way ANOVA analysis. Error bars represent the SD. *p < 0.05, **p < 0.01, ***p < 0.001, ****p < 0.0001

Co-culturing with 4T1 significantly upregulated the FAP expression in BALB/3T3 (Supplemental Fig. 2). Therefore, coculture-preconditioned BALB/3T3 fibroblasts were used in the subsequent experiment. EdU staining revealed that the combination of [^177^Lu]Lu-DOTAGA.(SA.FAPi)_2_ and AMD3100 suppressed the proliferation of BALB/3T3 cells at the greater extent than either monotherapy on days 1, 3, and 5 after treatment (Fig. 3). This trend was in line with the colony formation results, where the BALB/3T3 cells treated with combination therapy formed the fewest colonies among all groups (Supplemental Fig. 3). The proliferation of 4T1 cells was suppressed by AMD3100 or the combination treatment, but not by [^177^Lu]Lu-DOTAGA.(SA.FAPi)_2_ (Supplemental Fig. 4). The migration of 4T1, on the other hand, was blunted by AMD3100, [^177^Lu]Lu-DOTAGA.(SA.FAPi)_2_, or the combination, while the combination was more effective than either monotherapy (Supplemental Fig. 5).

### Therapeutic efficacy of the combination regime in mice

**Fig. 4:**
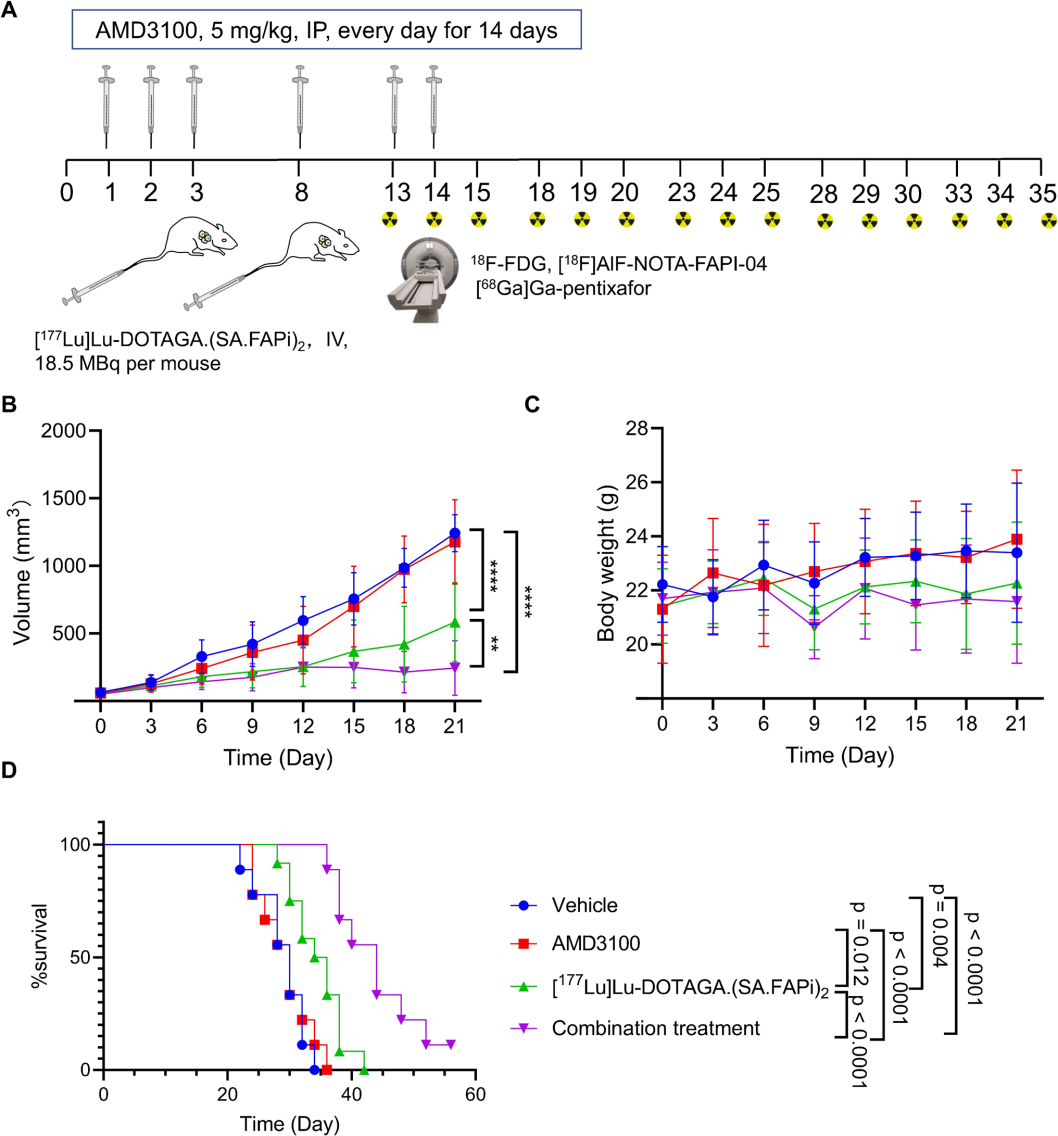
Therapeutic efficacy of AMD3100 and [^177^Lu]Lu-DOTAGA.(SA.FAPi)_2_ in 4T1 tumor model. **A** Treatment schedule and dosages. AMD3100 was intraperitoneally injected daily at 5 mg/kg/injection for 2 weeks. [^177^Lu]Lu-DOTAGA.(SA.FAPi)_2_ was intravenously injected at 18.5 MBq/injection on days 3 and 8. PET/CT scans with the three tracers were performed on days 13-15, 18-20, 23-25, 28-30, and 33-35. **B** Tumor growth curves, group size: vehicle (*n* = 9), AMD3100 (*n* = 9), [^177^Lu]Lu-DOTAGA.(SA.FAPi)_2_ (*n* = 10), combination treatment (*n* = 9). **C** Body weight change of 4T1 model suffered different treatment. **D** Kaplan–Meier survival curves of 4T1 model in groups of vehicle control (*n* = 9), AMD3100 (*n* = 9), [.^177^Lu]Lu-DOTAGA.(SA.FAPi)_2_ (*n* = 10), combination treatment (*n* = 9). Data in **B** and **C** are presented as mean ± SD in line diagram, statistical difference was evaluated using one‐way ANOVA analysis, and survival difference in (**D**) was evaluated using Log‐rank test. *p < 0.05, **p < 0.01, ***p < 0.001, ****p < 0.0001

The therapeutic efficacy of [^177^Lu]Lu-DOTAGA.(SA.FAPi)_2_ and AMD3100 was then investigated in the 4T1 xenograft model (Fig. 4A). Tumors treated with [^177^Lu]Lu-DOTAGA.(SA.FAPi)_2_ grew at a slower rate than those of vehicle controls. While AMD3100 alone did not suppressed tumor growth, the combination group exhibited greater tumor suppression than [^177^Lu]Lu-DOTAGA.(SA.FAPi)_2_. Accordingly, mice in the combination group exhibited significantly longer survival than the other groups (Fig. 4B, D). Mice treated with [^177^Lu]Lu-DOTAGA.(SA.FAPi)_2_ or the combination therapy experienced transient weight loss during the active treatment, but soon recovered without other discernable symptoms (Fig. 4C).

### Imaging

**Fig. 5:**
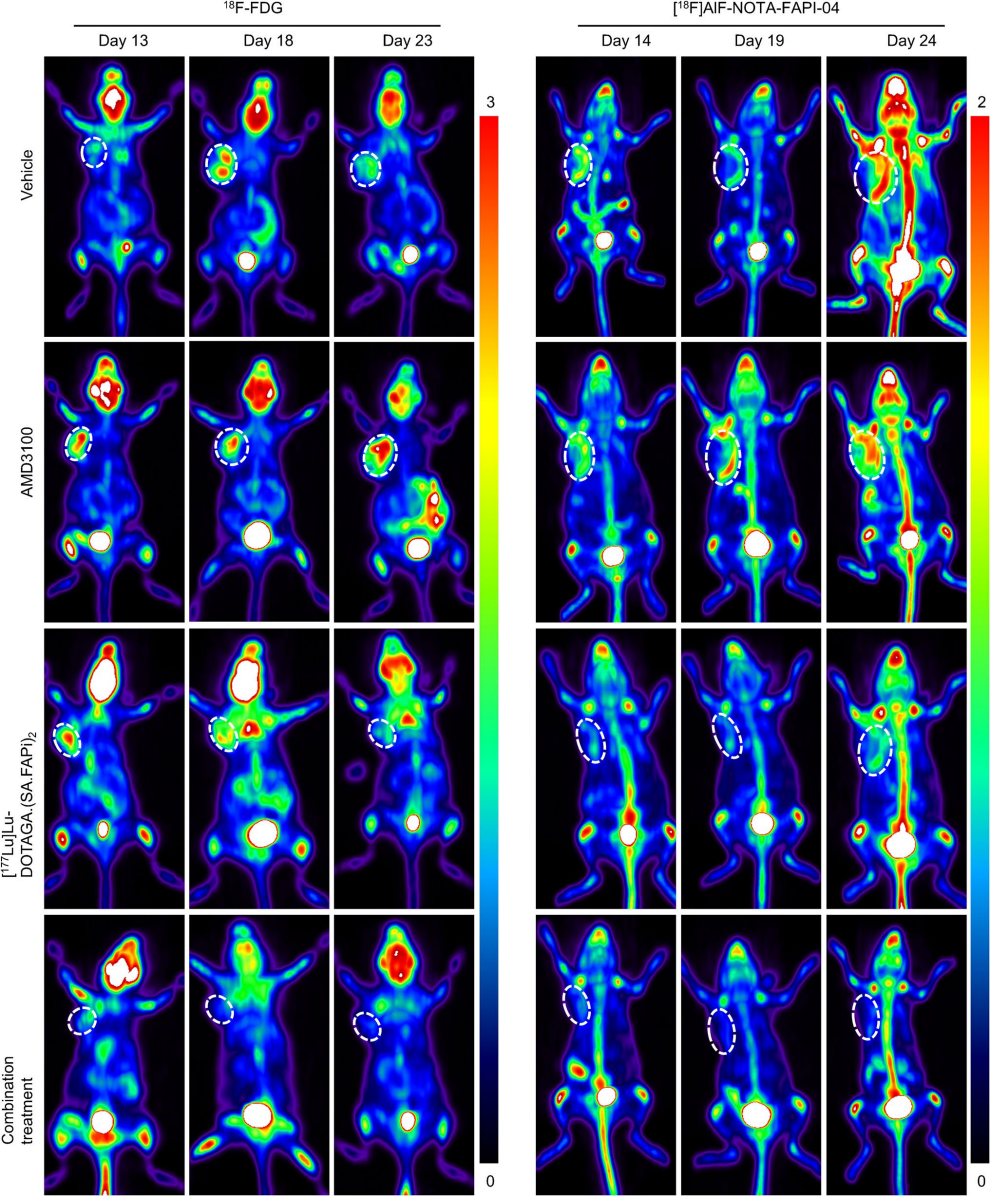
^18^F-FDG and [^18^F]AlF-NOTA-FAPI-04 PET/CT imaging during and after treatment. ^18^F-FDG and [^18^F]AlF-NOTA-FAPI-04 PET/CT imaging were performed on days 13-14, 18-19, and 23-24, respectively, from the initiation treatment (*n* = 4). 4T1-bearing mice were treated with vehicle, [^177^Lu]Lu-DOTAGA.(SA.FAPi)_2_, AMD3100, or combination of [^177^Lu]Lu-DOTAGA.(SA.FAPi)_2_ and AMD3100. Tumors were indicated by the white circle

^18^F-FDG, [^18^F]AlF-NOTA-FAPI-04, and [^68^ Ga]Ga-DOTA-Pentixafor all exhibited favorable capacity to visualize tumor lesions (Supplemental Fig. 6A). [^68^ Ga]Ga-DOTAGA.(SA.FAPi)_2_ exhibited durable tumoral retention (Supplemental Fig. 6B), consistent with its biodistribution data of [^177^Lu]Lu-DOTAGA.(SA.FAPi)_2_ [19]. Tumor uptake can also be clearly visualized 24 h after injection of [^177^Lu]Lu-DOTAGA.(SA.FAPi)_2_ (Supplemental Fig. 6C). Tumor-bearing mice in the AMD3100 group had the highest uptake of ^18^F-FDG among all the treatment groups on days 13, while the combination group had significantly lower uptake of ^18^F-FDG than the vehicle control. At later time points, ^18^F-FDG uptake in the AMD3100 treatment group was still higher than those in the [^177^Lu]Lu-DOTAGA.(SA.FAPi)_2_ or the combination groups. A similar trend was observed in the [^18^F]AlF-NOTA-FAPI-04 PET/CT imaging (Fig. 5, Supplemental Fig. 8A). No difference was observed in the [^68^Ga]Ga-DOTA-Pentixafor PET/CT imaging of all four groups.

Mice in the [^177^Lu]Lu-DOTAGA.(SA.FAPi)_2_ monotherapy and combination treatment group were further monitored using the three PET/CT tracers for up to 35 days after enrollment. Taken together, [^18^F]AlF-NOTA-FAPI-04 accumulation in both groups increased gradually following the conclusion of treatment regimens. The combination group had higher [^68^Ga]Ga-DOTA-Pentixafor uptake than the [^177^Lu]Lu-DOTAGA.(SA.FAPi)_2_ group starting from day 20. ^18^F-FDG uptake was similar in both groups at all the time points (Supplemental Fig. 7, Supplemental Fig. 8B).

### Immunohistochemistry

**Fig. 6:**
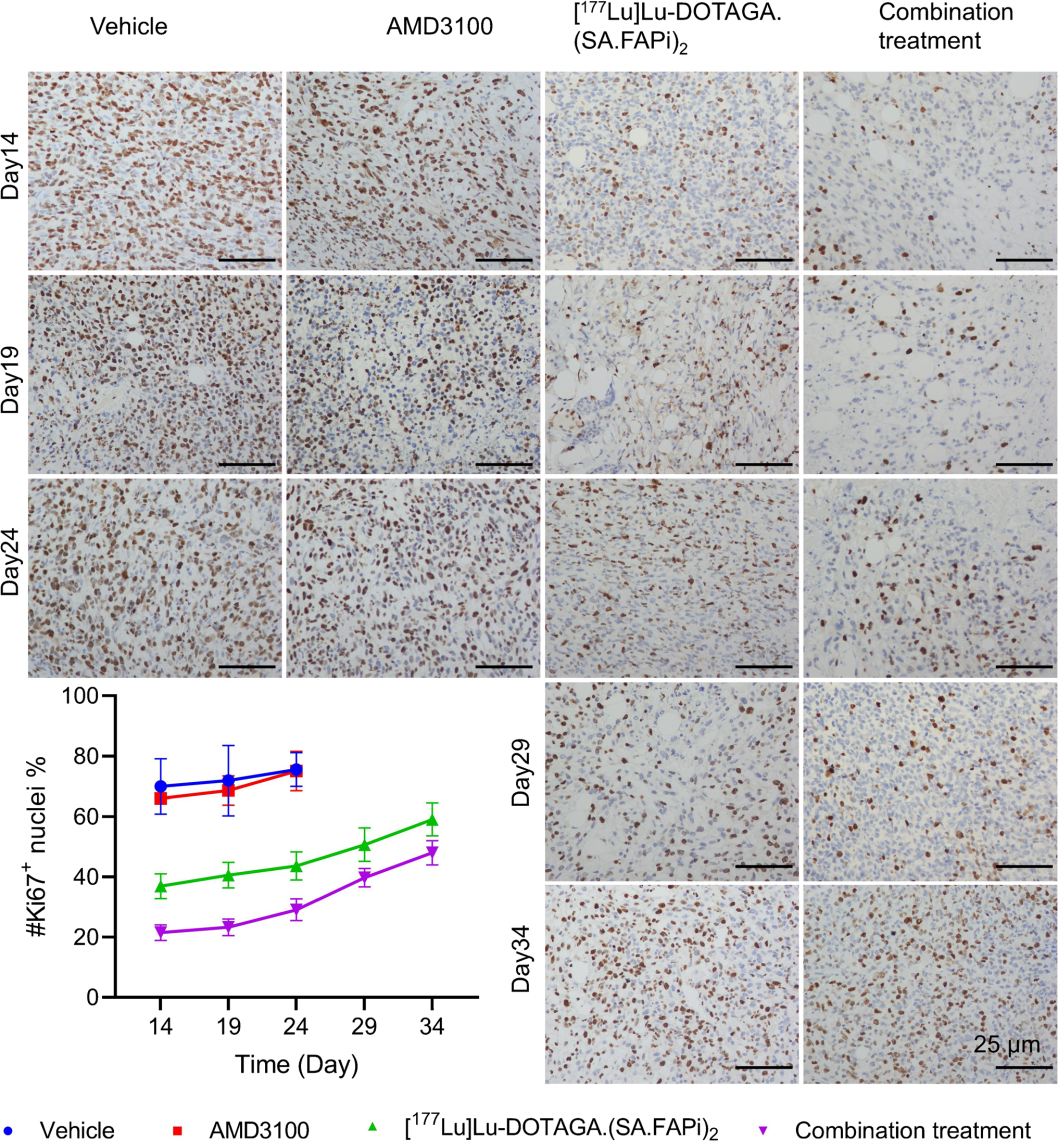
Tumoral immunohistochemical staining of Ki-67 on days 14, 19, 24, 29, and 34 from the initiation treatment. Mice were treated with vehicle control, [^177^Lu]Lu-DOTAGA.(SA.FAPi)_2_, AMD3100, or combination of both. The percentage of Ki-67 positive nuclei was quantified by ImageJ software and presented as mean ± SD in line diagram. Scale bar = 25 µm

Ki-67, α-SMA, CXCR4, and CXCL12 immunostaining were performed according to the PET/CT imaging schedule. Although the percentage of Ki-67^+^ tumor cells gradually increased over time, the combination group had the lowest abundancy of Ki-67^+^ tumor cells at all time points, indicating a sustained tumor suppression. Similar trends were observed in α-SMA, CXCR4, and CXCL12 staining (Fig. 6, Supplemental Fig. 9-12).

### Flow cytometry

**Fig. 7:**
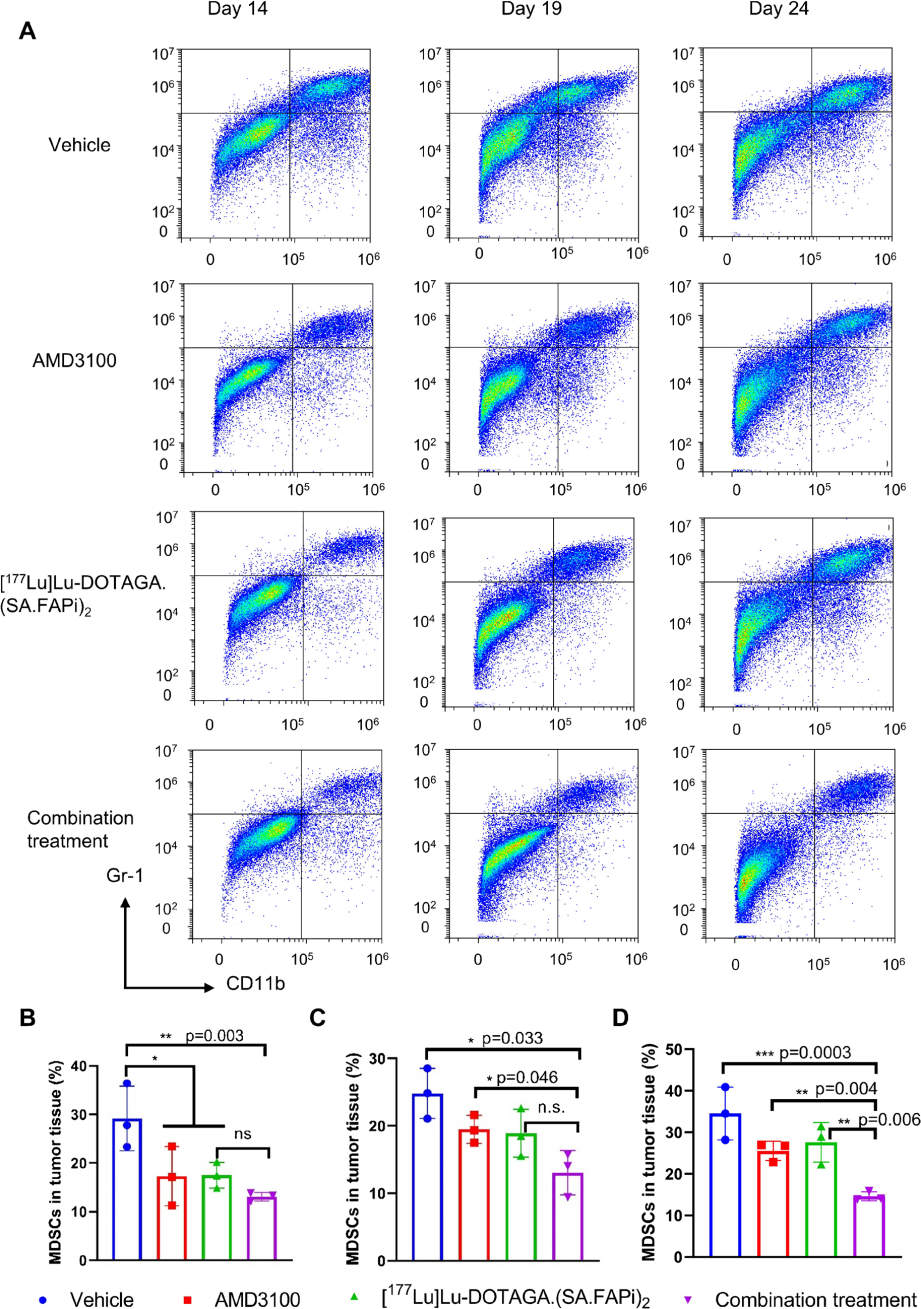
Representative flow cytometry images (**A**) and quantification of MDSCs’ numbers (CD11b^+^Gr-1^+^) (**B**-**D**). Tumor tissues were collected from mice of untreated control, AMD3100- or [^177^Lu]Lu-DOTAGA.(SA.FAPi)_2_-treated, or combination of both on days 14 (**B**), 19 (**C**), and 24 (**D**) from the initiation the treatment (*n* = 3). The percentage of MDSCs’ numbers in total tumoral single cell suspension was quantified. Significance of differences was determined using one‐way ANOVA analysis. *p < 0.05, **p < 0.01, ***p < 0.001, ****p < 0.0001

The dynamic changes in the numbers of MDSCs during and after treatment were monitored via flow cytometry (Fig. 7). Compared to vehicle control, the infiltration of MDSCs was reduced in the AMD3100 and [^177^Lu]Lu-DOTAGA.(SA.FAPi)_2_ monotherapy groups. The combination group had the lowest abundancy of MDSCs on all time points.

### Toxicity analysis

H&E staining of heart, lung, kidney, liver, and spleen was performed to investigate whether the combination therapy induced toxic reactions in major organs. Staining revealed no pathological alterations in all these organs (Supplemental Fig. 13).

## Discussion

In this study, we investigated the efficacy of combining [^177^Lu]Lu-DOTAGA.(SA.FAPi)_2_ and CXCR4 antagonist AMD3100 against the 4T1 murine breast tumor model. Our results demonstrated an enhanced and durable response in the combination treatment. We also evaluated the capability of ^18^F-FDG, [^18^F]AlF-NOTA-FAPI-04, and [^68^Ga]Ga-DOTA-Pentixafor PET/CT imaging to monitor therapeutic efficacy after treatment and explored the potential therapeutic mechanism.

Previous studies on CXCR4/CXCL12 signaling demonstrated that the interplay between CAFs and tumor cells played a critical role in tumor progression. There is also evidence that FAP-positive CAFs are the only stromal source of CXCL12. Therefore, blocking CXCR4/CXCL12 axis is a promising approach to treat TNBC. In this study, we administrated the [^177^Lu]Lu-DOTAGA.(SA.FAPi)_2_ to kill CAFs and surrounding tumor cells, resulting in a reduced secretion of CXCL12 (Supplemental Fig. 10). AMD3100 was administered to disrupt further CXCR4/CXCL12 interaction from the tumoral autocrine signaling (Supplemental Fig. 14). AMD3100 also reduced the infiltration of MDSCs (Fig. 7), consistent with earlier reports that AMD3100 remodeled the immunosuppressive TME [20].

We firstly verified that CAFs’ scores were negatively correlated with patient prognosis (Fig. 1), indicating that CAF is a viable target in the treatment of TNBC. Previous studies have identified four subsets of CAFs in breast cancer (CAF-S1 to CAF-S4); CAF-S1 was associated with an immunosuppressive TME and preferentially detected in TNBC, it is also the only subset of CAF with increased FAP expression [21]. There was a marked upregulation of CXCR4 and FAP in the tumor tissues of TNBC patients, suggesting that they promote tumor progression in a concerted manner. As documented earlier, CXCL12 secreted from FAP^+^ CAFs activated the CXCR4/CXCL12 cascade and exerted protumoral function in nearly every phases of TNBC [22]. Together, our results suggest that CAF depletion in conjunction with CXCR4 antagonists might be a promising approach for the treatment of TNBC.

In vitro, AMD3100 enhanced the efficacy of [^177^Lu]Lu-DOTAGA.(SA.FAPi)_2_ via suppressing fibroblast proliferation and 4T1 migration (Fig. 3 and Supplemental Fig. 4). Colony formation assay also revealed that AMD3100 further decreased the number of fibroblast colonies in the presence of [^177^Lu]Lu-DOTAGA.(SA.FAPi)_2_. We observed that [^177^Lu]Lu-DOTAGA.(SA.FAPi)_2_ monotherapy only marginally suppressed 4T1 proliferation. A possible explanation may be that [^177^Lu]Lu-DOTAGA.(SA.FAPi)_2_ could accumulate in conditioned BALB/3T3 fibroblasts more efficiently than in 4T1 cells [19]. Taken together, these data suggested that AMD3100 could inhibit both 4T1 cancer cells and BALB/3T3 fibroblasts from growth and migration in vitro. Our results also indicate that the recent therapeutic success of treatment of TNBC patients by a ^177^Lu-labelled FAPI homodimer could even be further improved by adding AMD3100 [16].

In 4T1 murine TNBC models, AMD3100 enhanced the efficacy of [^177^Lu]Lu-DOTAGA.(SA.FAPi)_2_ (Fig. 4). ^18^F-FDG PET/CT images (Fig. 5A) revealed a significantly lower SUVmax in the combination group than in the vehicle group. IHC data revealed a marked decreased in α-SMA staining in the [^177^Lu]Lu-DOTAGA.(SA.FAPi)_2_ and the combination groups, indicating a successful depletion of CAFs. The Ki-67 staining was also diminished in the two groups, supporting the tumoricidal potency of [^177^Lu]Lu-DOTAGA.(SA.FAPi)_2_ hat in both treatment groups (Fig. 6, Supplemental Fig. 9-12). There was no significant difference of Ki-67 staining between the two groups (*p* > 0.05), suggesting that AMD3100 did not directly enhance the tumoricidal potency of [^177^Lu]Lu-DOTAGA.(SA.FAPi)_2_. Indeed, unlike other radiosensitizers, AMD3100 is not known to disrupt the DNA damage response [19, 20]. The uptake of [^18^F]AlF-NOTA-FAPI-04 in the [^177^Lu]Lu-DOTAGA.(SA.FAPi)_2_ and the combination treatment groups was significantly lower than that in the control- and AMD3100-treated group, which could be attributed to the CAFs’ depletion by [^177^Lu]Lu-DOTAGA.(SA.FAPi)_2_. The addition of AMD3100 reduced [^18^F]AlF-NOTA-FAPI-04 uptake to a greater extent, especially on the days 14. It should be noted that the uptake of [^18^F]AlF-NOTA-FAPI-04 in these two groups increased gradually after the last treatment, indicating a continuously increasing number of CAFs, which is consistent with the IHC data (Supplemental Fig. 7). The uptake of [^68^Ga]Ga-DOTA-Pentixafor was significantly higher in the [^177^Lu]Lu-DOTAGA.(SA.FAPi)_2_ than in combination treatment on days 14. This pattern was reversed at later time points, however, which can be attributed to the compensatory CXCR4 upregulation as well as the tumor infiltration by CXCR4-expressing immune cells [23–25]. Minimal toxicity in main organs including heart, lung, kidney, liver, and spleen was recorded (Supplemental Fig. 13), indicating negligible side effects of this combination regime.

Our study has the following limitations. First, only 4T1 model was examined due to the limited supply of ^177^Lu. Second, this study only focused on the MDSCs, while other immunosuppressive immune cells like tumor-associated macrophages and tumor-associated neutrophils were not quantified. Third, DOTAGA.(SA.FAPi)_2_ can be further optimized to enhance its hydrophilicity, affinity, and selectivity, as exemplified by the recently explored DOTAGA.Glu.(FAPi)_2_ [26]. Lastly, this combination strategy only incompletely suppressed tumor lesion with a limited time frame. Gradual upregulated expression of Ki-67, α-SMA, CXCR4, and CXCL12 and increased numbers of MDSCs were observed after the conclusion of treatments (Figs. 6 and 7, Supplemental Fig. 9-12).

## Conclusion

The tumor-permissive and immunosuppressive characteristics of CXCR4/CXCL12 axis have fueled interest in therapeutically targeting this signal pathway. We herein established that the combination of CXCR4 antagonist AMD3100 could elevate therapeutic efficacy of [^177^Lu]Lu-DOTAGA.(SA.FAPi)_2_ via inhibiting cellular proliferation and migration in vitro, and reducing immunosuppressive MDSCs in vivo, with acceptable toxicity profiles. This strategy might provide a new treatment method to TNBC, and further studies will be undertaken to optimize it.

### Supplementary Information

Below is the link to the electronic supplementary material.

Supplementary file1 (DOCX 6131 KB)

## Data Availability

The datasets generated during and/or analyzed during the current study are available from the corresponding author on reasonable request.
