# Supplementary material for: Targeting CXCR4/CXCL12 axis via [177Lu]Lu-DOTAGA.(SA.FAPi)2 with CXCR4 antagonist in triple-negative breast cancer
Source: Eur J Nucl Med Mol Imaging. 2024 Apr 8;51(9):2744–57. doi: 10.1007/s00259-024-06704-y (PMC11224082; doi:10.1007/s00259-024-06704-y)
Supplement: Supplementary file 1 — Supplementary file1 (DOCX 6131 KB) [file 259_2024_6704_MOESM1_ESM.docx]

**Title page**

**Targeting CXCR4/CXCL12 axis via** **[^177^Lu]Lu-DOTAGA.(SA.FAPi)_2_ with CXCR4 antagonist in Triple-negative Breast Cancer**

Guangfa Bao^1#^, Ziqiang Wang^1#^, Luoxia Liu^1^, Buchuan Zhang^1^, Shuang Song^1^, Dongdong Wang^1^, Siyuan Cheng^1^, Eu-Song Moon^2^, Frank Roesch^2^, Jun Zhao^1,3,4^, Bo Yu^1^, Xiaohua Zhu^1*^

**Affiliations****:**

^1^Department of Nuclear Medicine, Tongji Hospital, Tongji Medical College, Huazhong University of Science and Technology, Wuhan, China.

^2^Department of Chemistry, Johannes Gutenberg University, 55131 Mainz, Germany.

^3^Department of Anatomy, School of Basic Medicine, Huazhong University of Science and Technology, Hubei Province, Wuhan 430030, China.

^4^Cell Architecture Research Center, Huazhong University of Science and Technology, Hubei Province, Wuhan 430030, China.

^#^These authors contributed equally to this work (Guangfa Bao and Ziqiang Wang).

**Corresponding author：**

*Prof. Dr. Xiaohua Zhu, Department of Nuclear Medicine, Tongji Hospital, Tongji Medical College, Huazhong University of Science and Technology, Wuhan, China. Address: 1095 Jiefang Ave, Wuhan, 430030, China. Tel: +86-27-83663446. Fax: +86-27-83663446. Email: evazhu@vip.sina.com. ORCID: 0000-0003-0495-9510.

**Acknowledgments**

This work was supported by the National Natural Science Foundation of China (Nos. 82272041, 91959119, 81873903, 82001871).


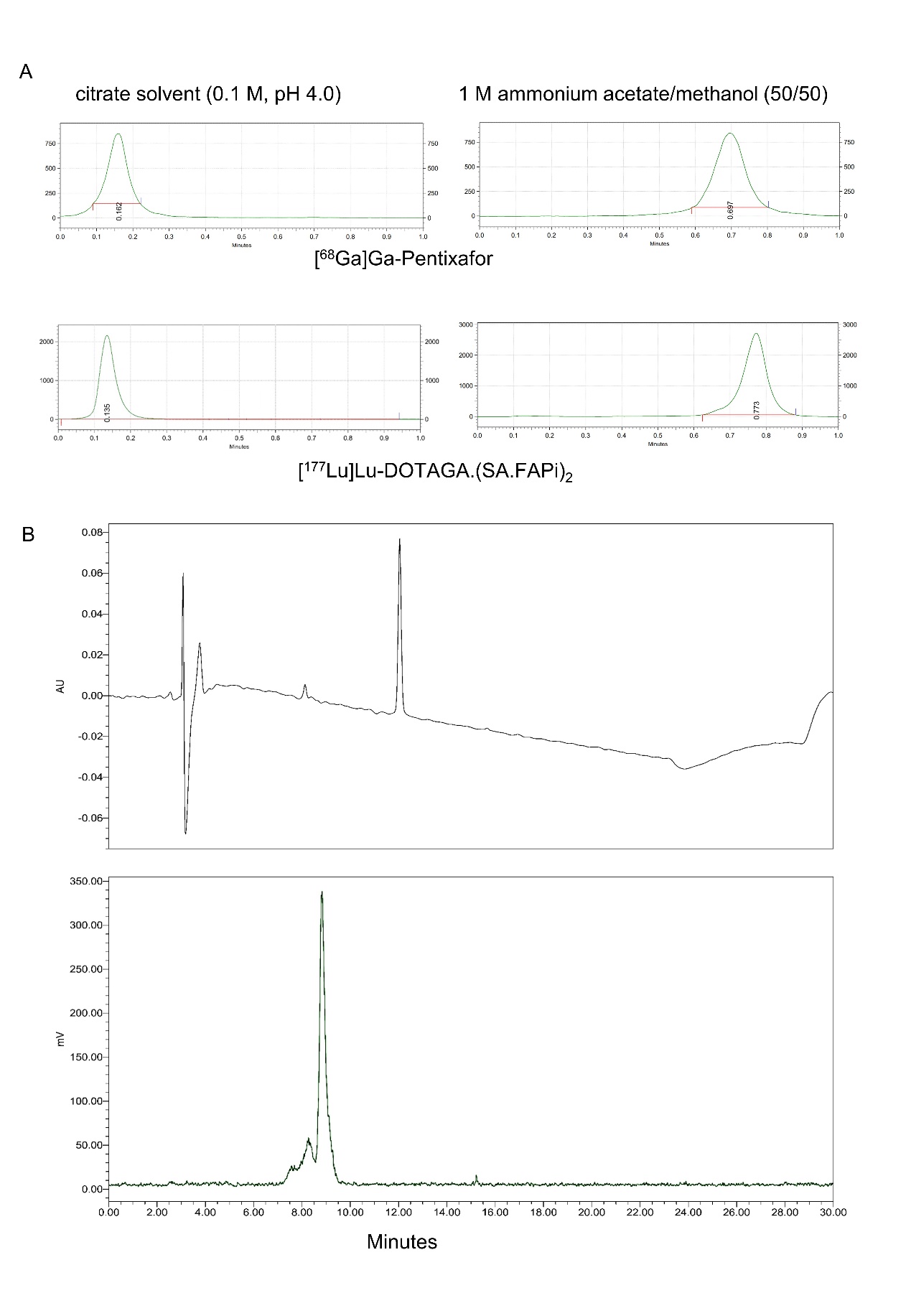


**Supplemental Fig. 1** **Quality control.** (A) Radiochemical purity for [^68^Ga]Ga-DOTA-Pentixafor and [^177^Lu]Lu-DOTAGA.(SA.FAPi)_2_ determined with ITLC method. (B) Characterization of [^18^F]AlF-NOTA-FAPI-04 via HPLC.


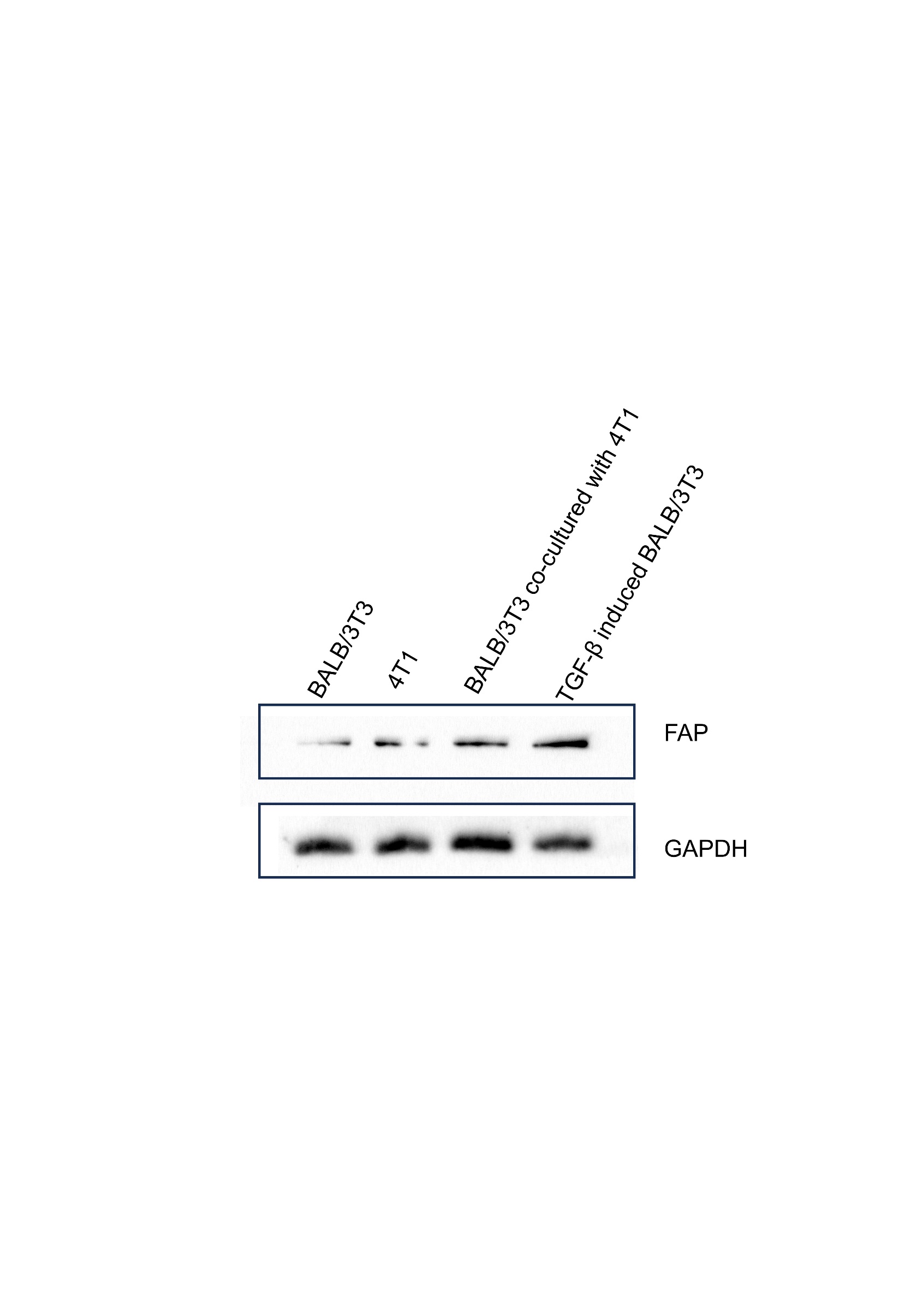


**Supplemental Fig. 2 Preliminary study to evaluate FAP expression.** BALB/3T3 and 4T1 cells were co-cultured indirectly with a transwell setting. TGF-β was added to a final concentration of 10 ng/mL and incubated with the BALB/3T3 for 48 h.


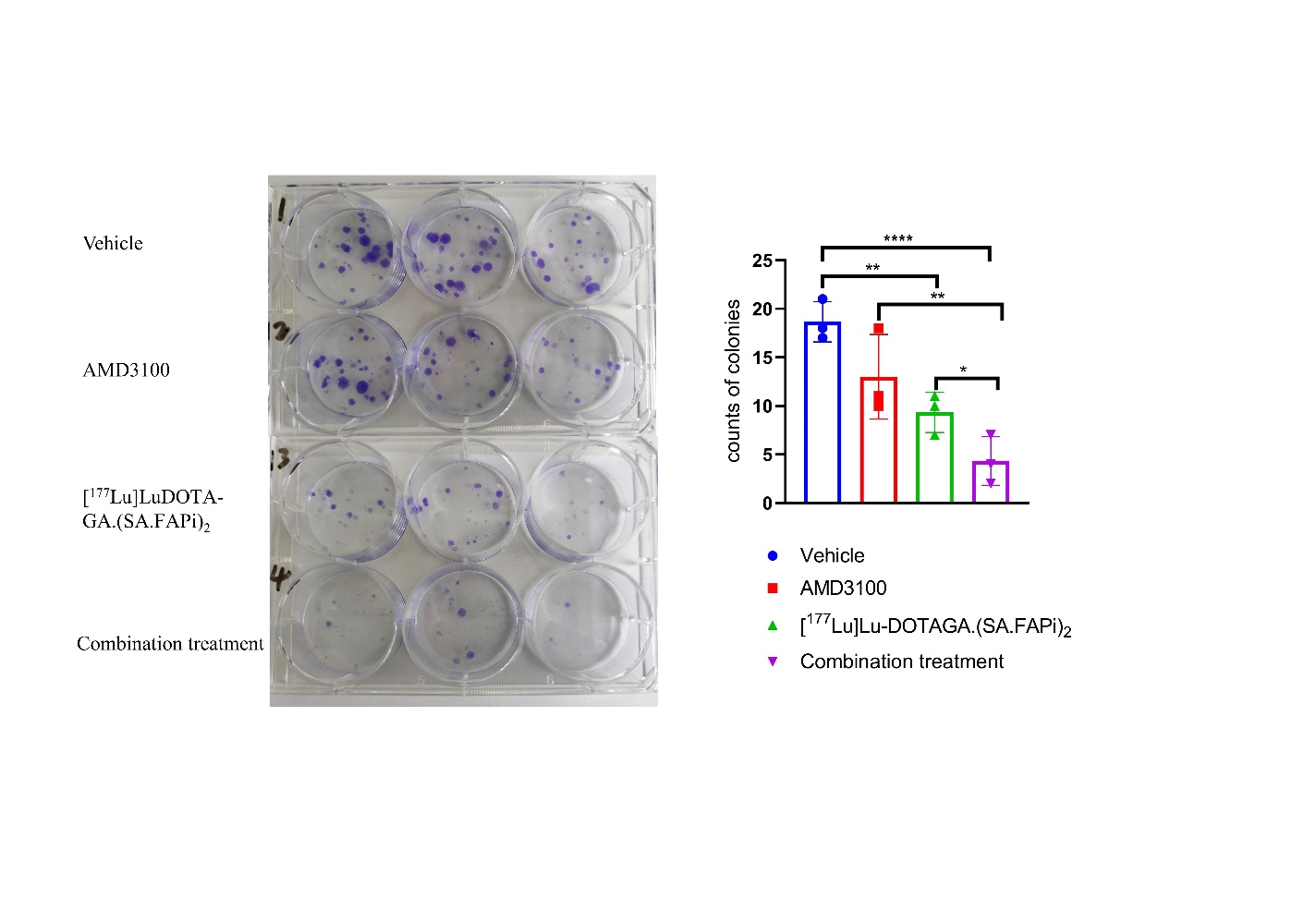


**Supplemental Fig. 3** Effect of AMD3100 on the colony formation assay of BALB/3T3 fibroblast after [^177^Lu]Lu-DOTAGA.(SA.FAPi)_2_ treatment. **p* < 0.05, ***p* < 0.01, ****p* < 0.001, *****p* < 0.0001.


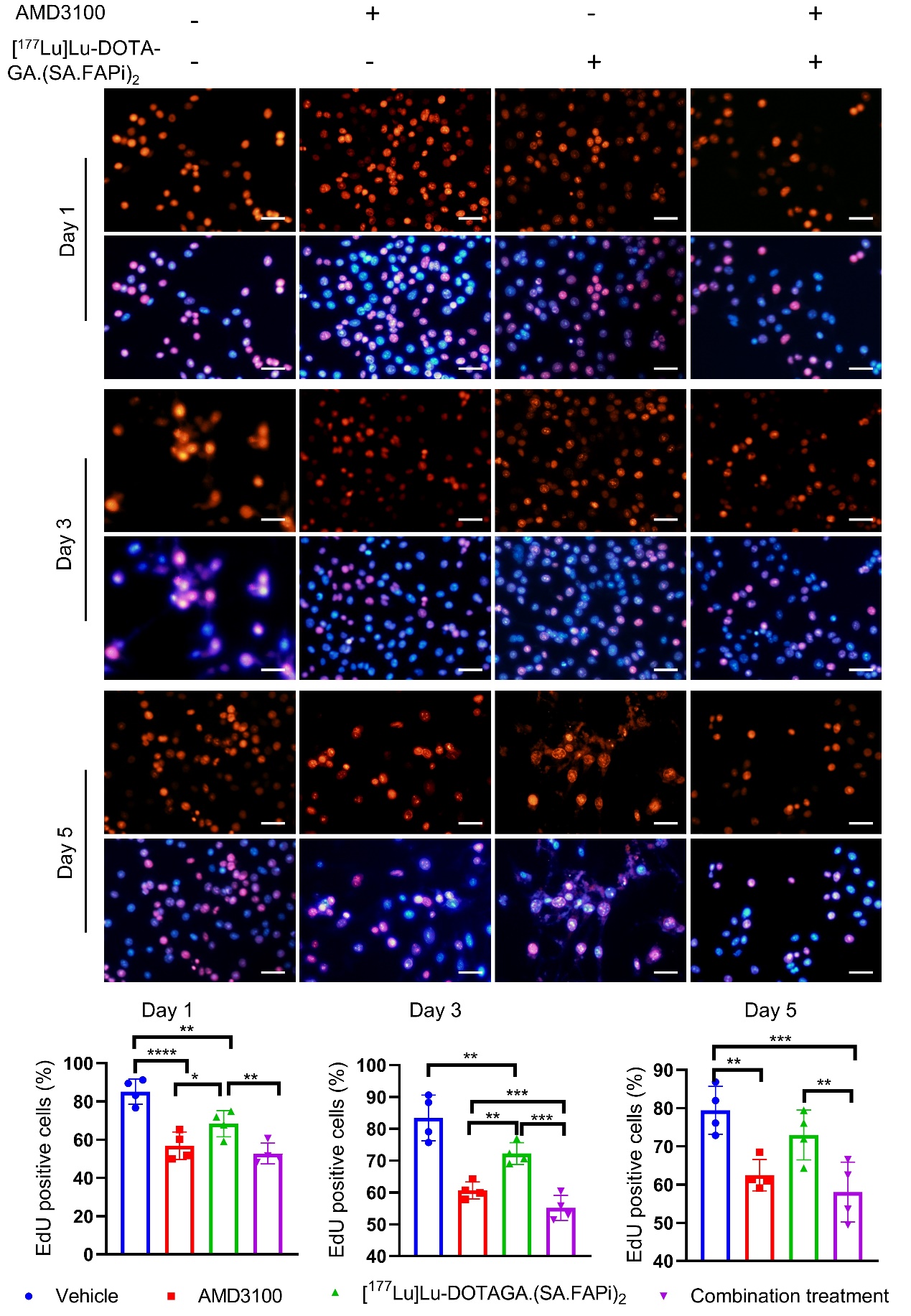


**Supplemental Fig. 4** **Effect of AMD3100 on 4T1 cell proliferation** **after [^177^Lu]Lu-DOTAGA.(SA.FAPi)_2_ treatment**. Immunofluorescent EdU (red) and Hoechst (blue) staining of 4T1 cells on days 1, 3, 5 after treatment. Cells were non-treated, treated with [^177^Lu]Lu-DOTAGA.(SA.FAPi)_2_, AMD3100, or combination of both. The percentage of EdU positive cells were quantified and analyzed using one‐way ANOVA analysis. Error bars represent the SD. **p* < 0.05, ***p* < 0.01, ****p* < 0.001, *****p* < 0.0001, scale bar = 25μm.


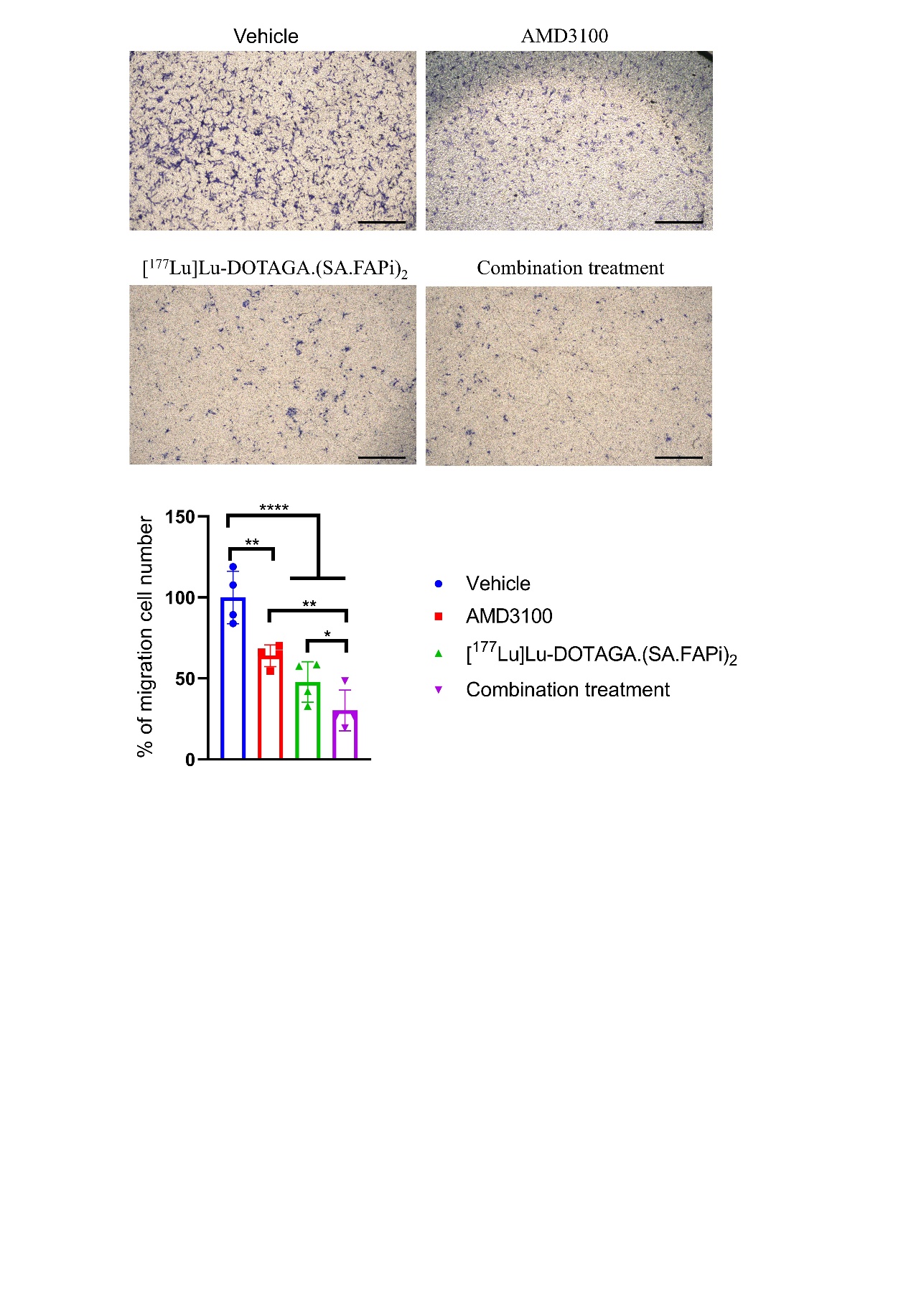


**Supplemental Fig. 5** **Effect of AMD3100 on the migration of 4T1 cells after [^177^Lu]Lu-DOTAGA.(SA.FAPi)_2_ treatment.** Cells were non-treated, treated with [^177^Lu]Lu-DOTAGA.(SA.FAPi)_2_, AMD3100, or combination of both. The numbers of migrated 4T1cells were quantified and analyzed using one‐way ANOVA analysis. Error bars represent the SD. **p* < 0.05, ***p* < 0.01, ****p* < 0.001, *****p* < 0.0001. Scale bar = 250 μm.


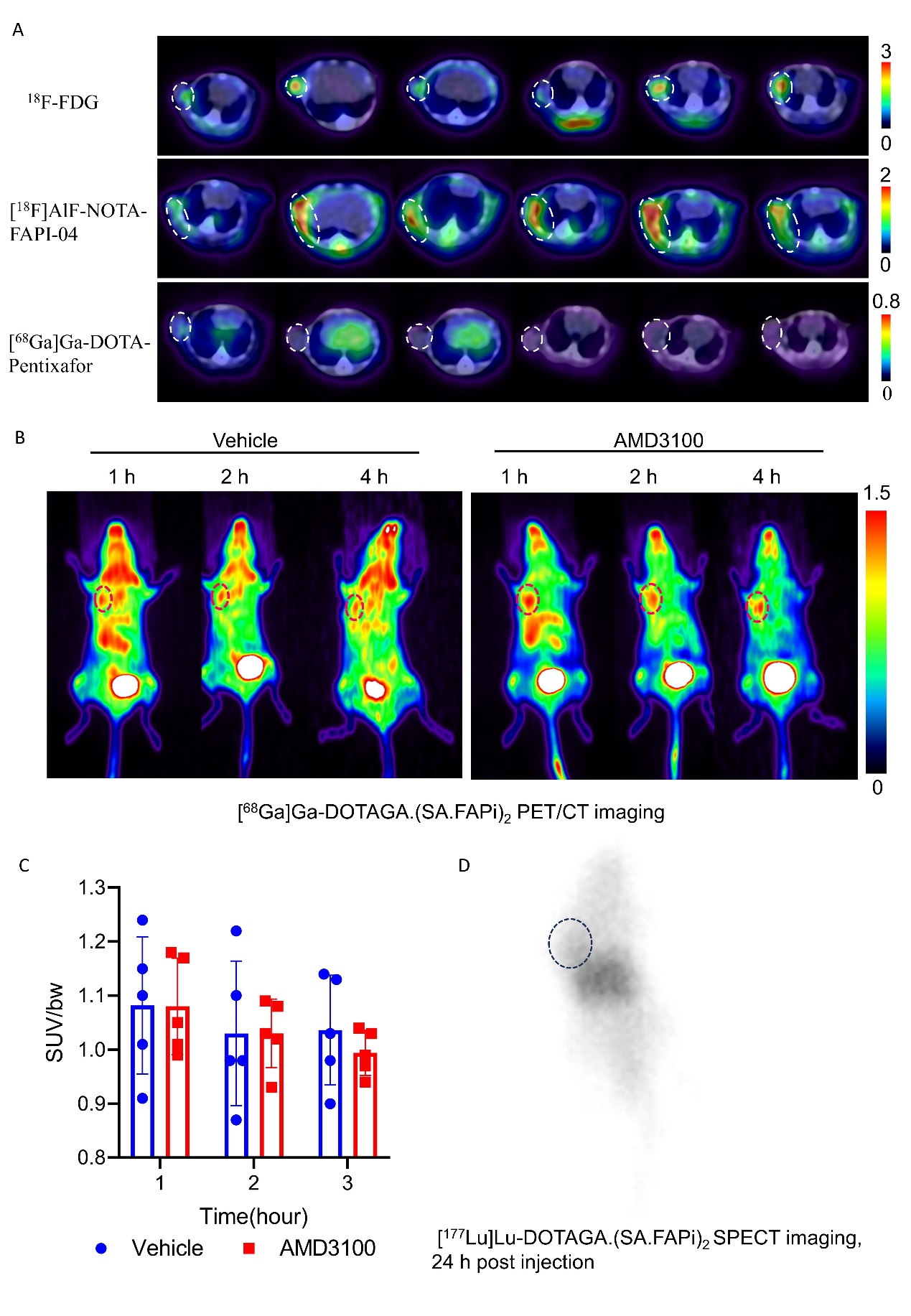


**Supplemental Fig. 6 Preliminary study of PET/CT imaging and SPECT imaging.** (A) ^18^F-FDG, [^18^F]AlF-NOTA-FAPI-04, [^68^Ga]Ga-DOTA-Pentixafor PET/CT imaging to evaluate the capacity of these tracers for imaging 4T1 tumors (n = 6). (B) [^68^Ga]Ga-DOTAGA.(SA.FAPi)_2_ PET/CT imaging in 4T1 bearing mice (n = 5), mice were treated daily with vehicle or AMD3100 for 3 days before imaging. Tumors were indicated by the red circles. (C) Quantification of tumoral accumulation of [^68^Ga]Ga-DOTAGA.(SA.FAPi)_2_, significance of differences was determined using Student t-test. No statistic difference was observed between these two groups, tumors were indicated by the red circles. (D) Representative [^177^Lu]Lu-DOTAGA.(SA.FAPi)_2_ SPECT imaging at 24 h post injection, tumor was indicated by the black circle.


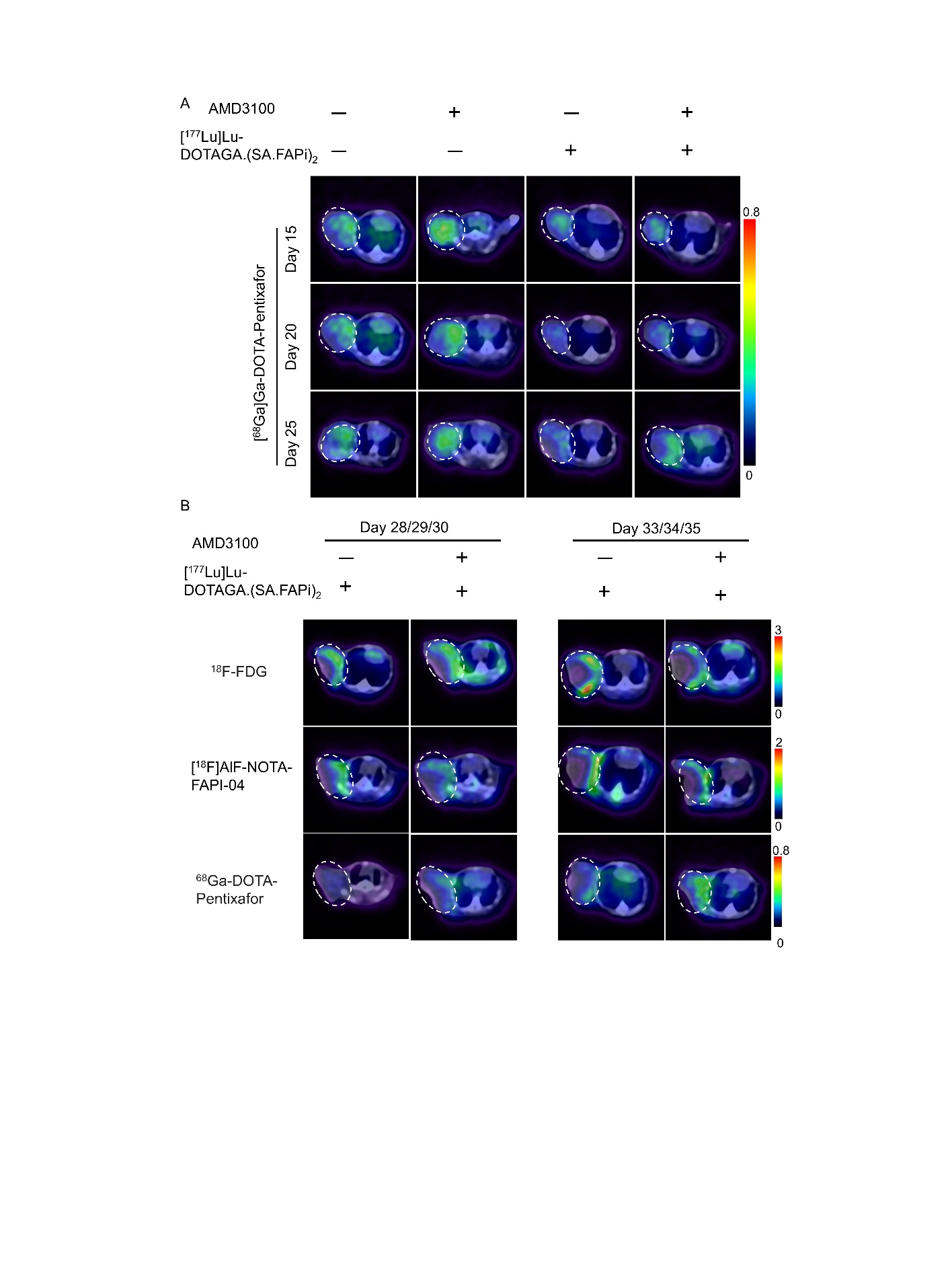


**Supplemental Fig. 7** **PET/CT imaging.** (A) [^68^Ga]Ga-DOTA-Pentixafor PET/CT imaging on days 15, 20, 25 from the initiation the treatment. Mice were received different therapy respectively according to their grouping (n = 4). (B) ^18^F-FDG, [^18^F]AlF-NOTA-FAPI-04, [^68^Ga]Ga-DOTA-Pentixafor PET/CT imaging of [^177^Lu]Lu-DOTAGA.(SA.FAPi)_2_ or combination treated mice on days 28-30, 33-35 from the initiation the treatment (n = 3) .


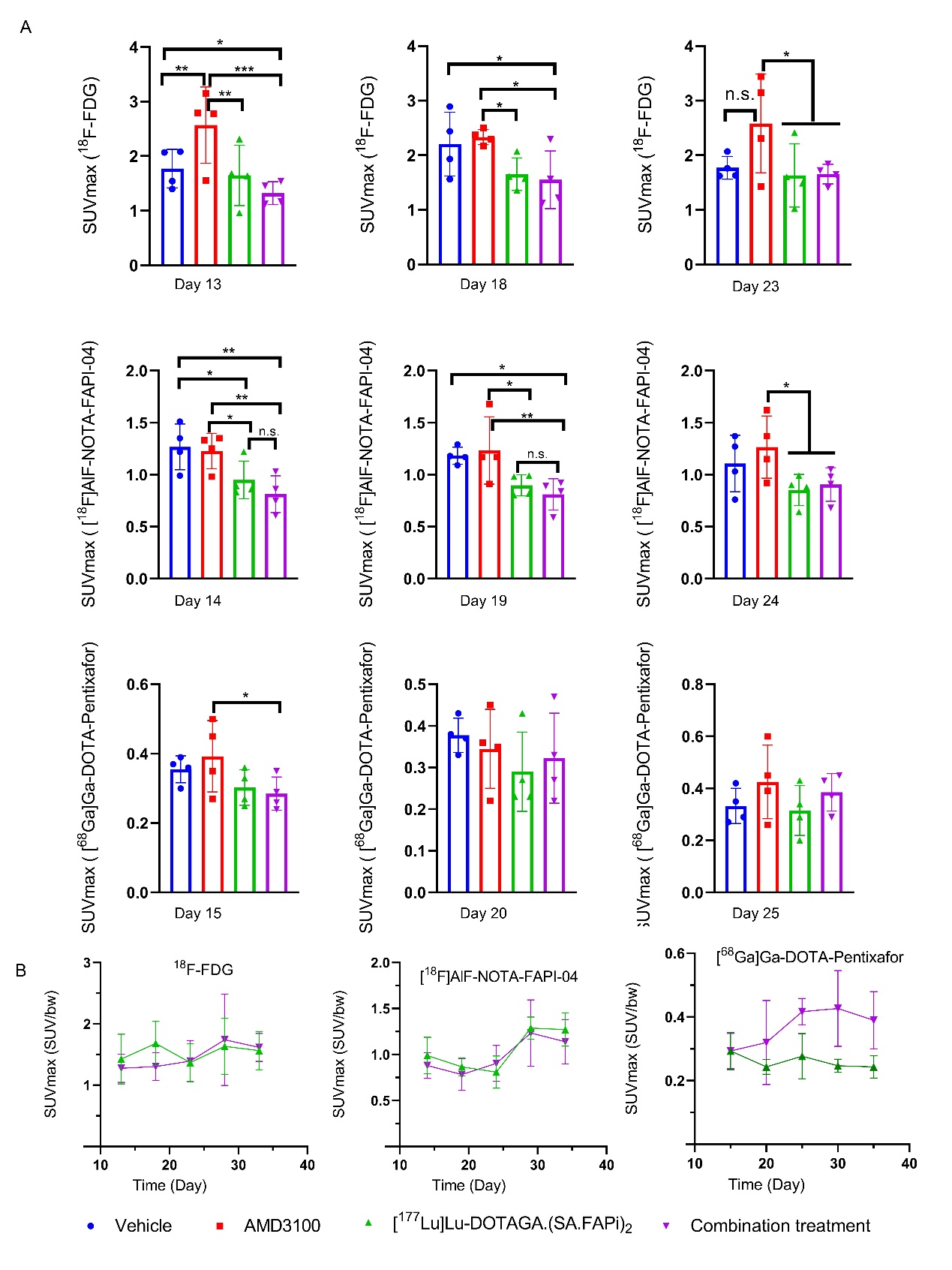


**Supplemental Fig. 8 Quantification of tracer uptake during and after treatment.** (A) quantification of tumoral accumulation of ^18^F-FDG, [^18^F]AlF-NOTA-FAPI-04, [^68^Ga]Ga-DOTA-Pentixafor on days 13-15, 18-20, 23-25 from the initiation the treatment (n = 4). Significance of differences was determined using one-way ANOVA analysis. **p* < 0.05, ***p* < 0.01, ****p* < 0.001. (B) Line diagram to depict the dynamic changes of SUVmax of three tracers in [^177^Lu]Lu-DOTAGA.(SA.FAPi)_2_- or combination treated mice at all PET/CT scans (n = 3).


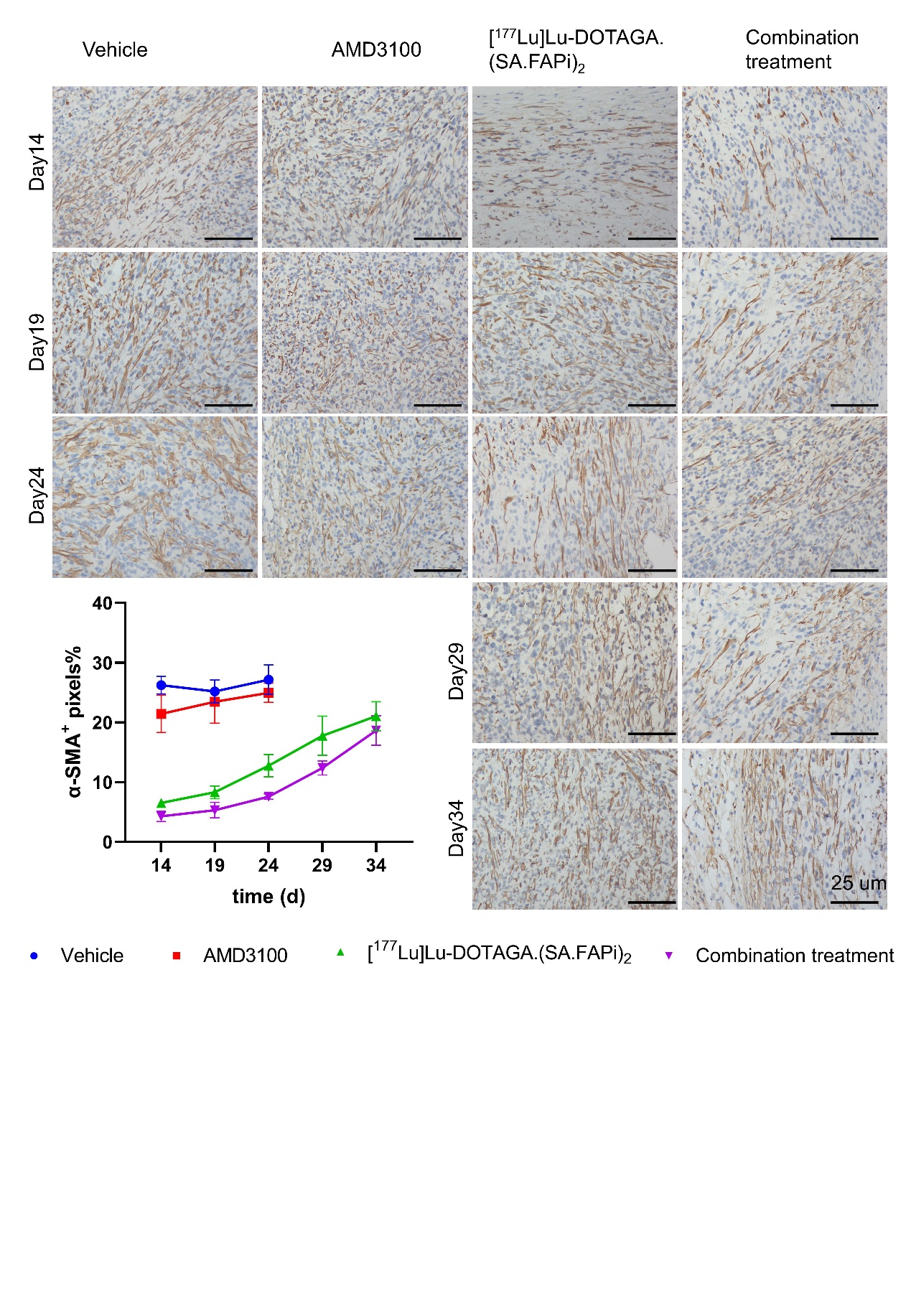


**Supplemental Fig. 9** Immunohistochemical staining (α-SMA) on days 14, 19, 24, 29, 34 from the initiation the treatment. Mice were treated with vehicle control, [^177^Lu]Lu-DOTAGA.(SA.FAPi)_2_, AMD3100, or combination of both. The percentage of α-SMA positive pixels was quantified and presented as mean ± SD in line diagram. Scale bar = 25 µm.


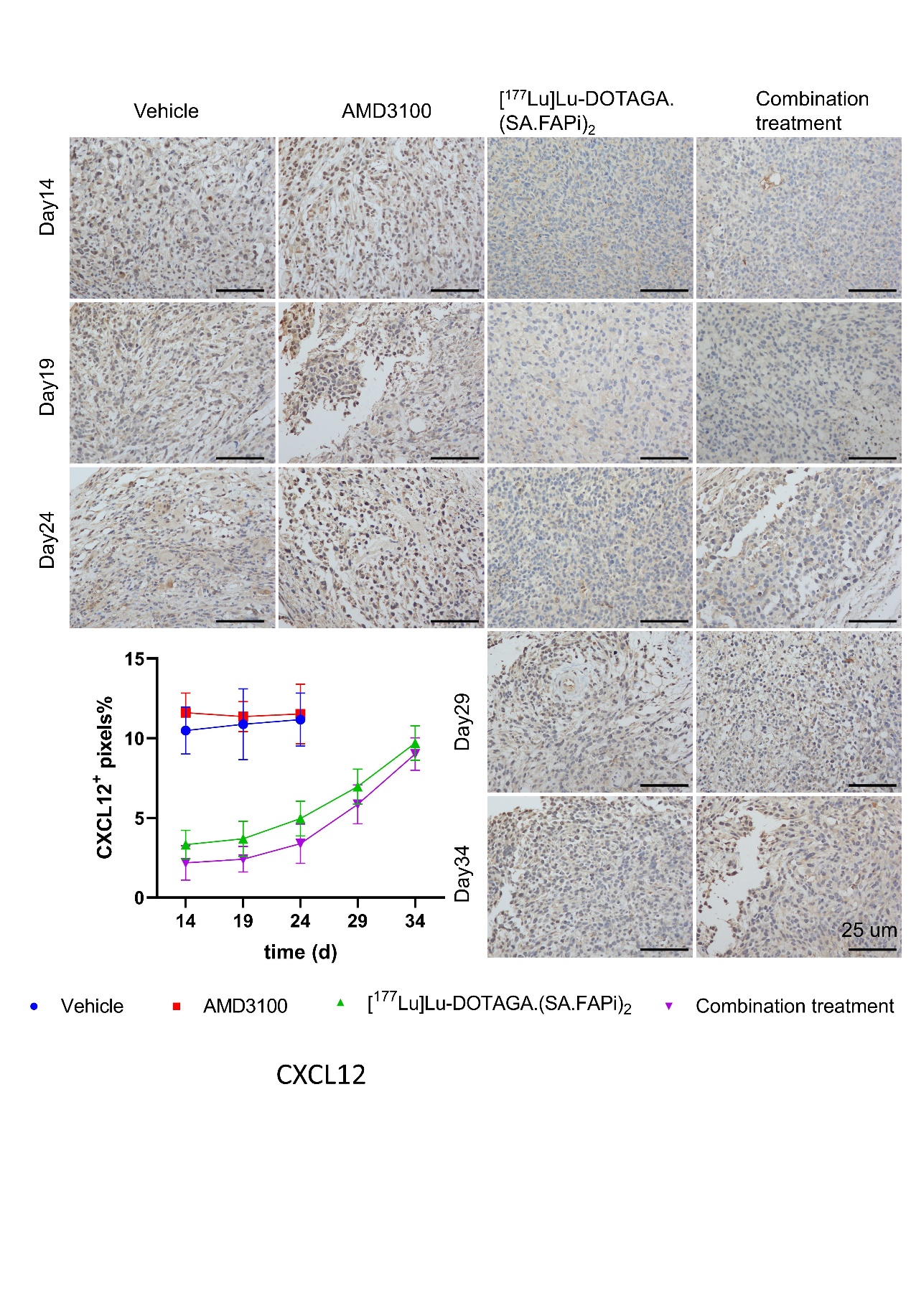


**Supplemental Fig. 10** **Immunohistochemical staining (CXCL12) on days 14, 19, 24, 29, 34 from the initiation the treatment.** Mice were treated with vehicle control, [^177^Lu]Lu-DOTAGA.(SA.FAPi)_2_, AMD3100, or combination of both. The percentage of CXCL12 positive pixels was quantified and presented as mean ± SD in line diagram. Scale bar = 25 µm.


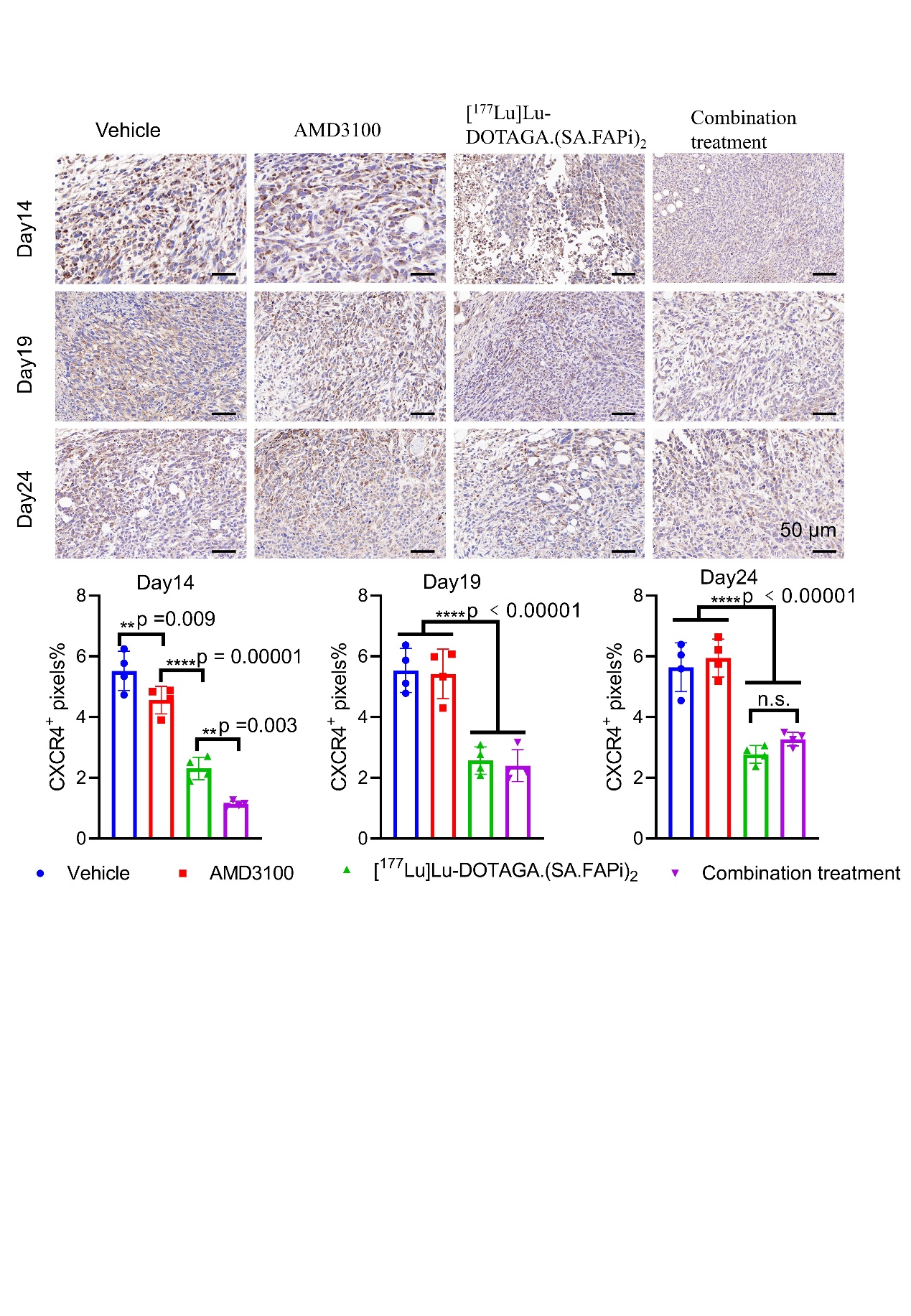


**Supplemental Fig. 11 Tumoral immunohistochemical staining (CXCR4) on days 14, 19, 24 from the initiation the treatment.** Mice were treated with vehicle control, [^177^Lu]Lu-DOTAGA.(SA.FAPi)_2_, AMD3100, or combination of both. The percentage of CXCR4 positive area was quantified by ImageJ software and presented as mean ± SD in bar diagram. Statistical difference was determined using one‐way ANOVA analysis. n.s. not significance, **p* < 0.05, ***p* < 0.01, ****p* < 0.001, *****p* < 0.0001, scale bar = 50 µm.


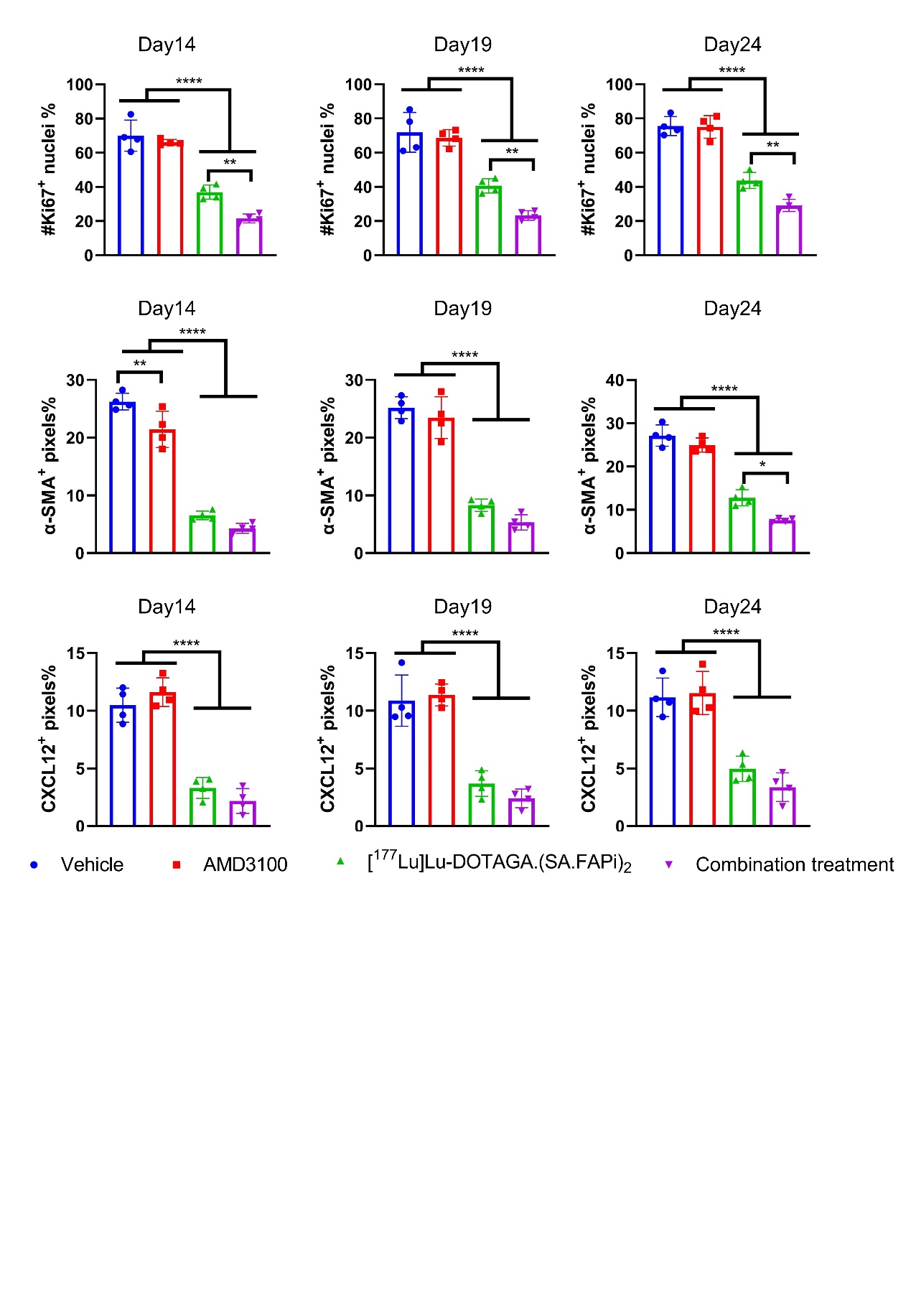


**Supplemental Fig. 12** **Respective quantification of IHC staining of Ki-67, α-SMA and CXCL12 on days 14, 19, 24** **from the initiation the treatment.** Mice were treated with vehicle control, [^177^Lu]Lu-DOTAGA.(SA.FAPi)_2_, AMD3100, or combination of both (n = 4). The percentage of positive nuclei/pixels were quantified by ImageJ software and presented as mean ± SD in bar diagram. Statistical difference was determined using one‐way ANOVA analysis **p* < 0.05, ***p* < 0.01, ****p* < 0.001, *****p* < 0.0001.


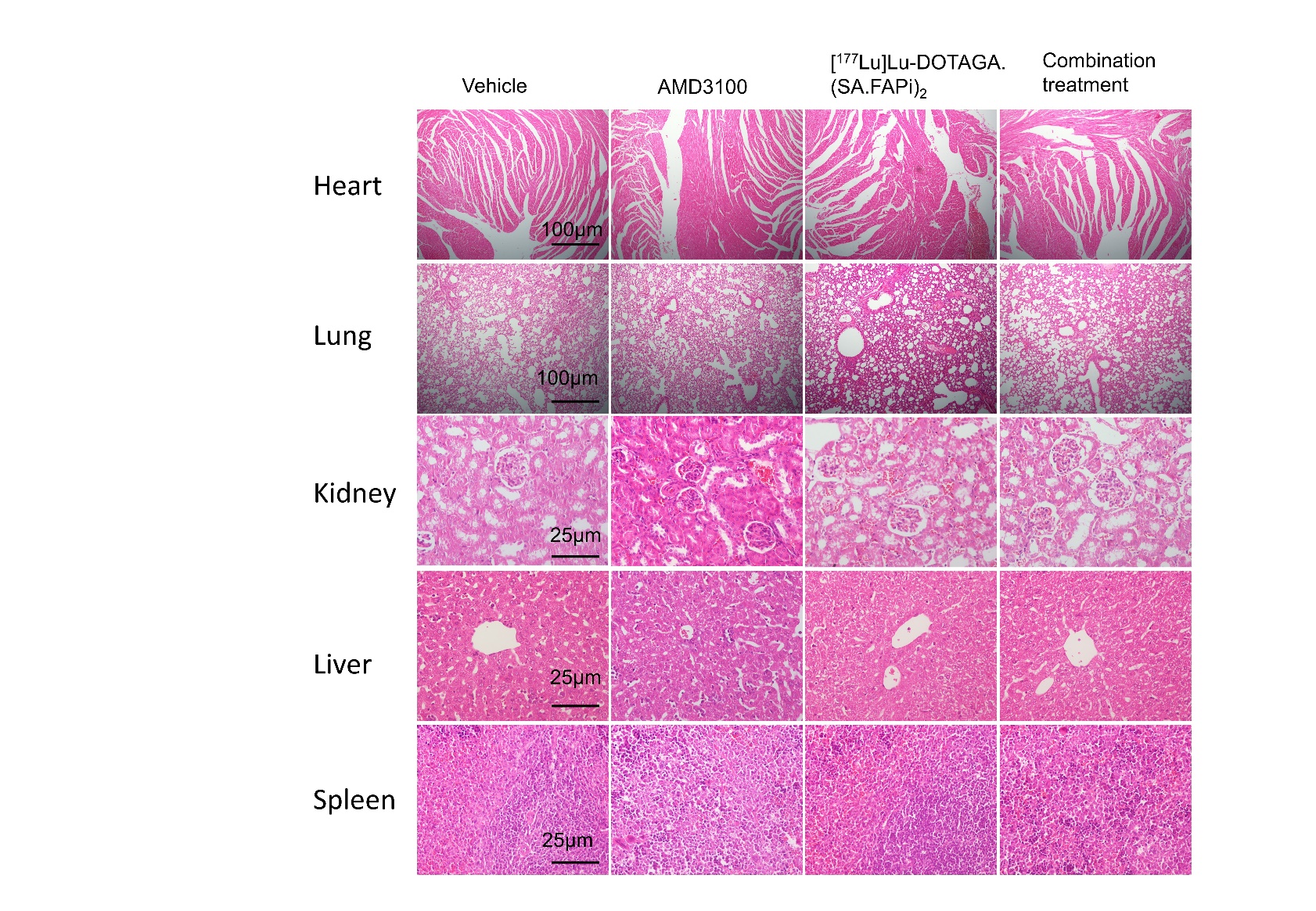


**Supplemental Fig. 13** **Toxicity analyses of [^177^Lu]Lu-DOTAGA.(SA.FAPi)_2_ + AMD3100 treatment.** Representative H&E staining of major organs including heart, lung, kidney, liver and spleen are presented. Mice were treated with vehicle control, [^177^Lu]Lu-DOTAGA.(SA.FAPi)_2_, AMD3100, or combination of both (n = 4). Organs were collected on days 19 from the initiation the treatment. The scale bar for heart and lung staining was 100 µm, and others were 25 µm.


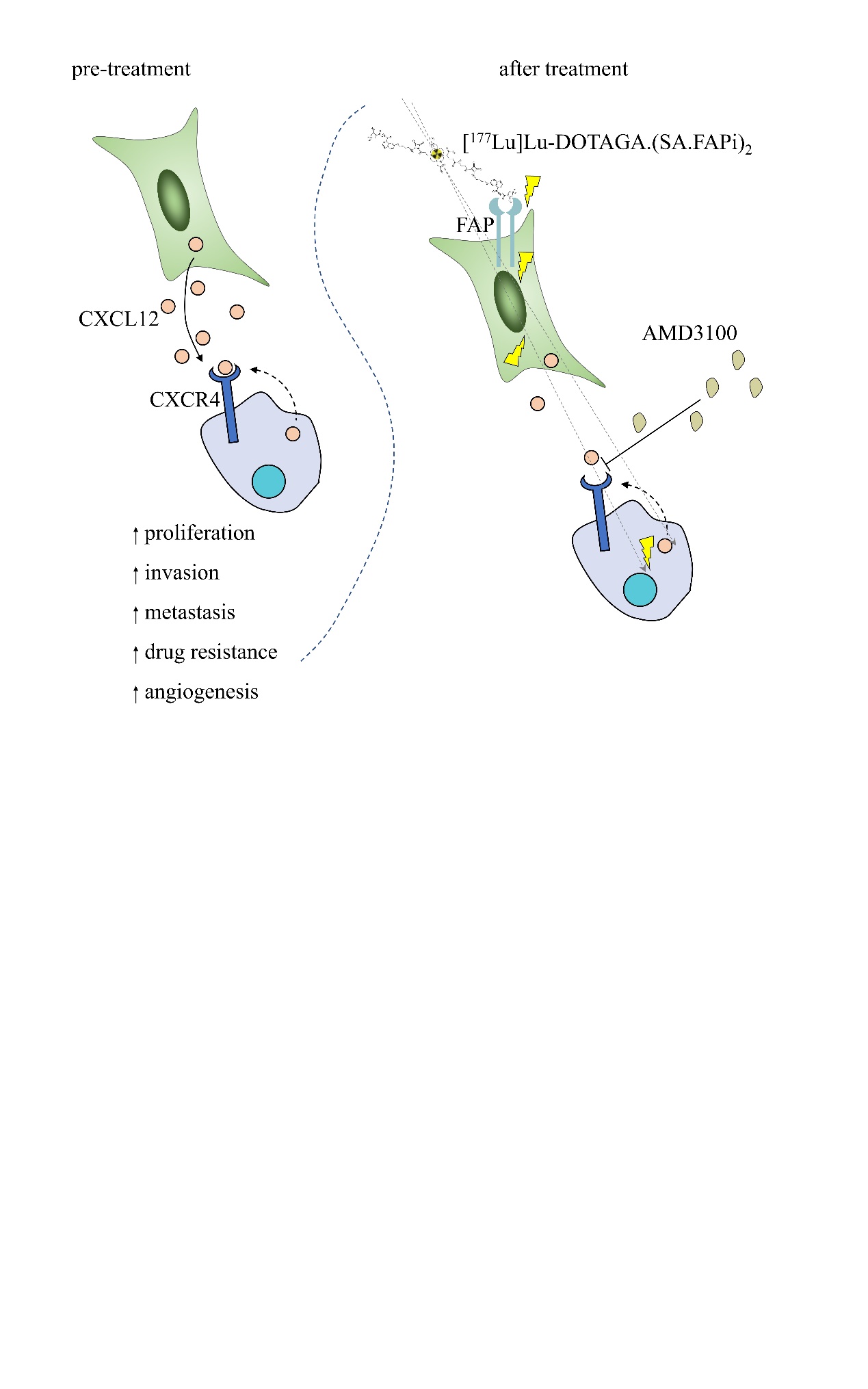


**Supplemental Fig. 14** **Schematic diagram of mechanism of the combination of [^177^Lu]Lu-DOTAGA.(SA.FAPi)_2_ and AMD3100 treatment.** [^177^Lu]Lu-DOTAGA.(SA.FAPi)_2_ specifically bind to the CAFs and kill them, reducing the secretion of CXCL12, the addition of AMD3100 not only directly enhance the therapeutic efficacy of [^177^Lu]Lu-DOTAGA.(SA.FAPi)_2_ but also inhibit the extra CXCR4/CXCL12 signaling such as the tumoral autocrine CXCL12.
